# Supplementary material for: Mobile phone data reveal the effects of violence on internal displacement in Afghanistan
Source: Nat Hum Behav. 2022 May 12;6(5):624–34. doi: 10.1038/s41562-022-01336-4 (PMC9130096; doi:10.1038/s41562-022-01336-4)
Supplement: Supplementary file 1 — Supplementary Figs. 1–8, Tables 1–9 and information on data processing. [file 41562_2022_1336_MOESM1_ESM.pdf]

nature portfolio

https://doi.org/10.1038/s41562-022-01336-4

Supplementary information

Mobile phone data reveal the effects of violence on internal displacement in Afghanistan

In the format provided by the authors and unedited

Supplementary Material for "Mobile Phone Data Reveal the Effects of Violence on Internal Displacement in Afghanistan"

Xiao Hui Tai[a], Shikhar Mehra[a], and Joshua E. Blumenstock[a,*]

[a]School of Information, University of California, Berkeley, CA 94720, USA
[*]Corresponding author: jblumenstock@berkeley.edu

This version of text:
March 4, 2022

Data Processing

We have access to anonymized mobile phone metadata from Afghanistan's largest mobile phone operator, during the period April 2013 to March 2017. We use metadata from call and internet access records. Each record contains a hash for the subscriber, the date and time, as well as cell tower the individual connected to. We have the geocoordinates of each cell tower. We group towers within 100 meters of each other into the same group, resulting in 1439 cell tower groups.

For each subscriber, we first determine the "hourly modal tower," which we define to be the most frequently used cell tower group in each hour in which data are available for that subscriber. Then for each 24-hour period from 6 A.M. to 6 A.M. the next day, we select the modal tower group based on hourly modal towers. We define this to be the "daily modal tower" of that subscriber on that day. We then map the daily modal tower to the district based on the location of tower, obtaining a "daily modal location" for each subscriber. This will be available for each subscriber on days in which they have at least one transaction (call or data use).

Next, we use daily modal locations to estimate each subscriber's residence or home location. To reiterate, daily locations may be sparse if subscribers do not use their phones every day, and might not accurately reflect a subscriber's residence; for example it might encode weekend trips. We use an open-source Python package, `migration_detector` [1], available at `https://github.com/g-chi/migration_detector`, to infer a longer-term residence. More details of the algorithm are available in the accompanying documentation and paper. Briefly, segments in time with contiguous locations are grouped to identify persistent changes in locations. The algorithm incorporates tuning parameters, which we select such that the resulting location segments roughly correspond to a subscriber being in a district for at least a week-long period. Specifically, we set `num_stayed_days_migrant, num_days_missing_gap, small_seg_len, seg_prop, min_overlap_part_len` to 5, 2, 7, 0.5 and 0 respectively.

We aggregate these individual home locations, inferred from `migration_detector`, to a district level, to derive the statistics used in the paper. These include the number of people per district per day, the number of people moving each day between pairs of districts, and so forth.

References

[1] Chi, G., Lin, F., Chi, G. & Blumenstock, J. A general approach to detecting migration events in digital trace data. PloS one **15**, e0239408 (2020).

[2] Sundberg, R. & Melander, E. Introducing the ucdp georeferenced event dataset. Journal of Peace Research **50**, 523–532 (2013).

[3] Çakıt, E. & Karwowski, W. Fuzzy inference modeling with the help of fuzzy clustering for predicting the occurrence of adverse events in an active theater of war. Applied Artificial Intelligence **29**, 945–961 (2015).

Supplementary Figures

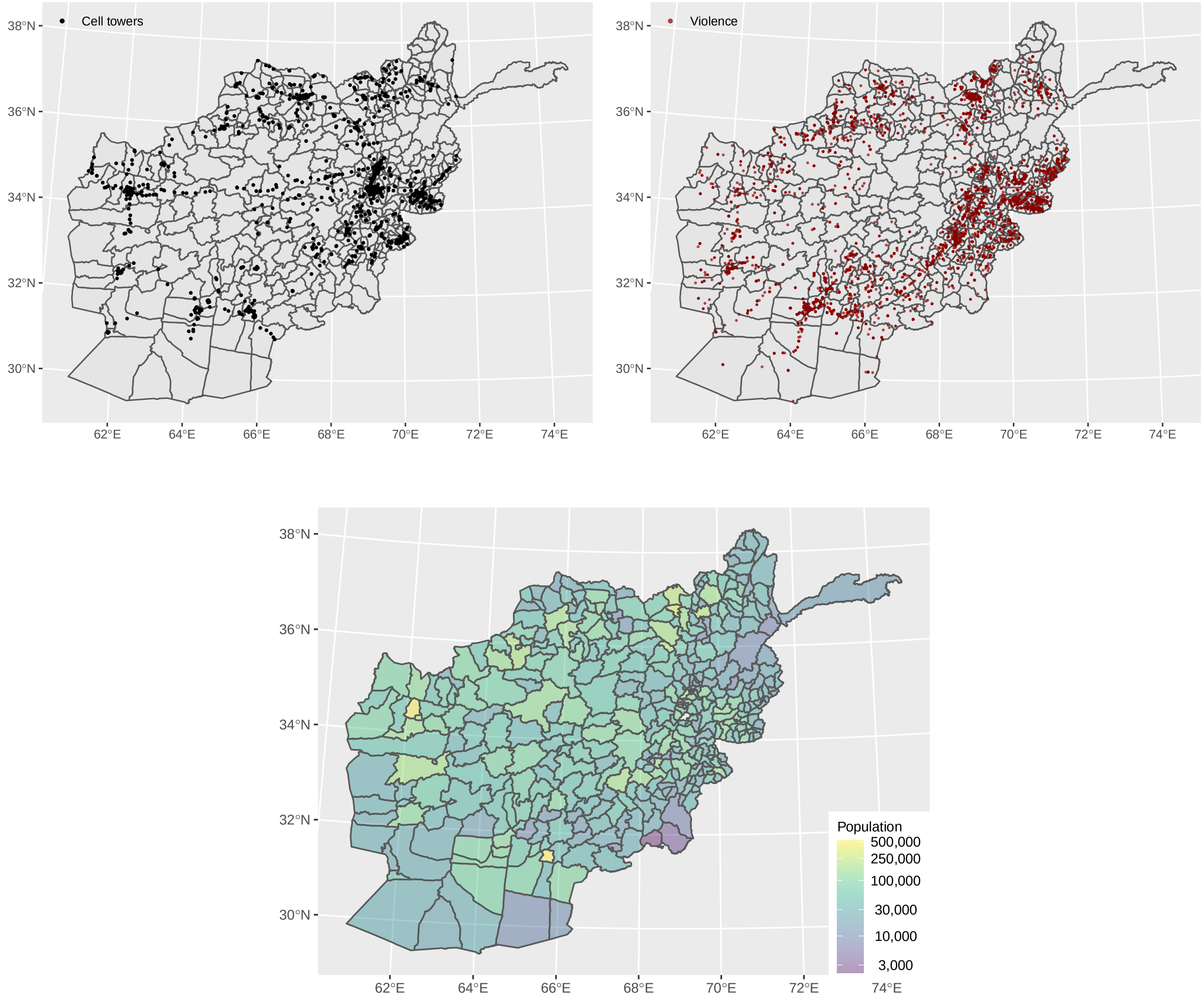

Supplementary Figure 1: Map of cell towers and violent events in Afghanistan, 2013-2017, and district-level population, 2015-2016. District boundaries are represented by black and gray lines. There are 1439 cell tower groups (black dots) and 5984 violent events (red dots) [2]. Event locations are marked by exact geocoordinates when available, or with the district centroid when only the district name is known. Population data is as of 2015-2016, available at `https://data.humdata.org/dataset/estimated-population-of-afghanistan-2015-2016`. Kabul is not colored as it distorts the scale; its recorded population is 3.7 million.

Supplementary Figure 2: Comparison of International Organization for Migration (IOM)-based estimates of displacement (official data) and Call Detail Records (CDR)-based estimates of migration (mobile phone data). IOM data uses the counts of recorded "fled IDPs (internally displaced persons)" for 2016 (outgoing IDPs; $N = 1209125$), and the number of "returnee" and "arrival" IDPs (incoming IDPs; $N = 2143281$). Blue bars represent each province's share of the total number of (A) outgoing and (B) incoming IDPs. Red bars represent province shares of outgoing and incoming migrants, computed using CDR. CDR migrants are those who have different recorded locations at the beginning and the end of three four-month periods in 2016 (Jan-Apr, Apr-Aug, and Aug-Dec; summed to obtain a measure of movement in 2016). The number of CDR migrants is scaled using district population as estimated by Afghanistan's Central Statistical Office, to get a district estimate of number of migrants (after scaling, $N = 5632789$ for outgoing migrants, and $N = 5654994$ for incoming migrants). Province shares are then computed in the same way as for official data. Kabul district is not included in the figure as it distorts the y-scale of the axes.

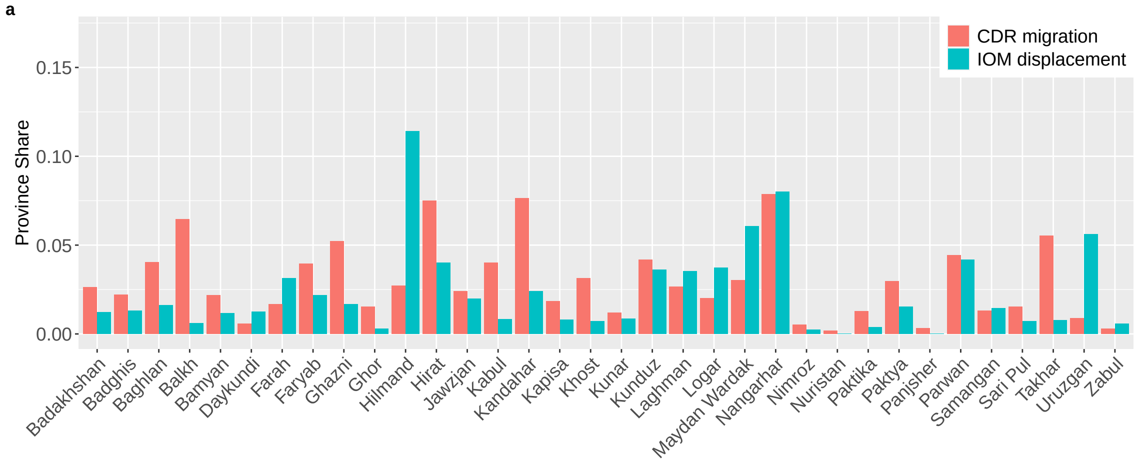

(a) Province shares of total outgoing individuals, by origin province.

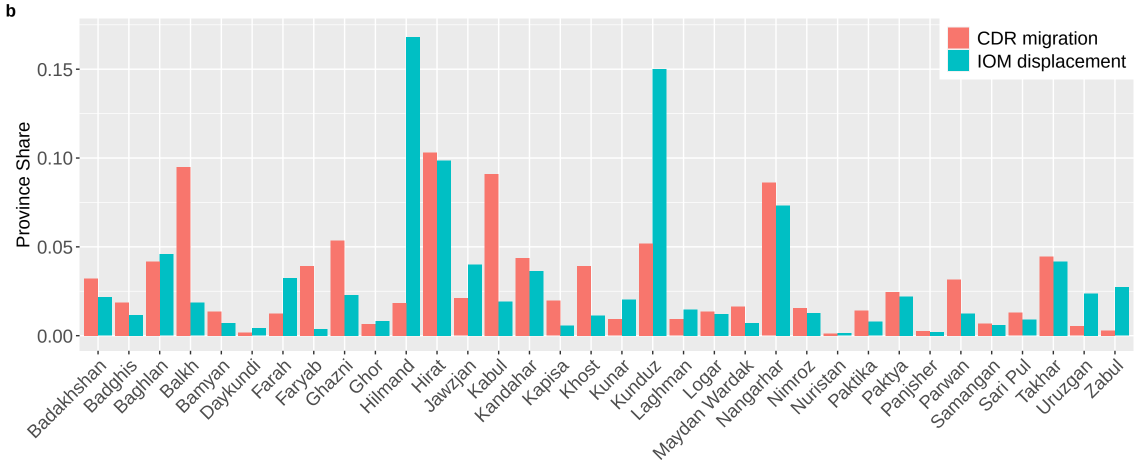

(b) Province shares of total incoming individuals, by destination province.

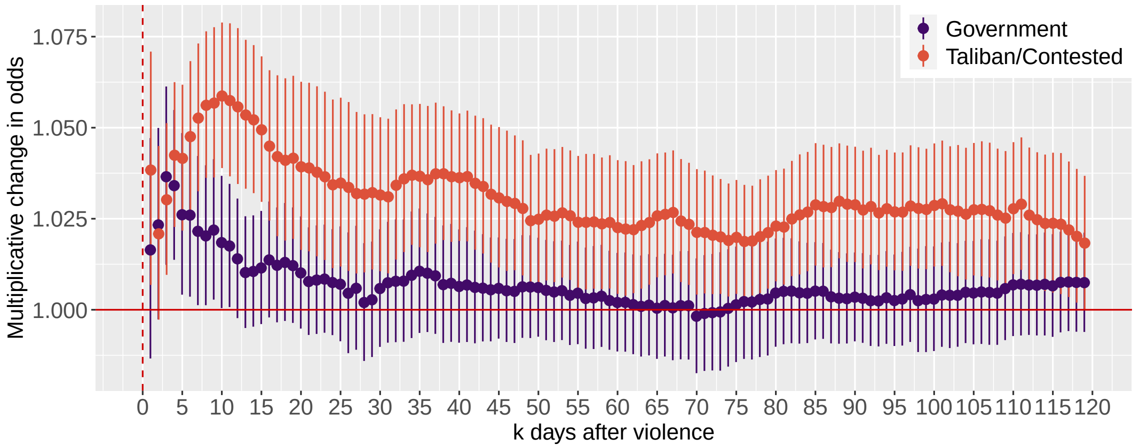

Supplementary Figure 3: Figure shows the effect of violence on internal displacement for events in districts under government control or influence (N=1469) vs. events in districts that are contested or under insurgent control or influence (N=1885). Territorial control is as of October 2017, assessed by the Special Inspector General for Afghanistan Reconstruction, and available at `https://www.sigar.mil/pdf/quarterlyreports/Addendum_2018-01-30qr.pdf`. By this measure, 221 districts are classified as government controlled or influenced, with the remaining 177 contested or under insurgent control or influence. Bars indicate 95% confidence intervals.

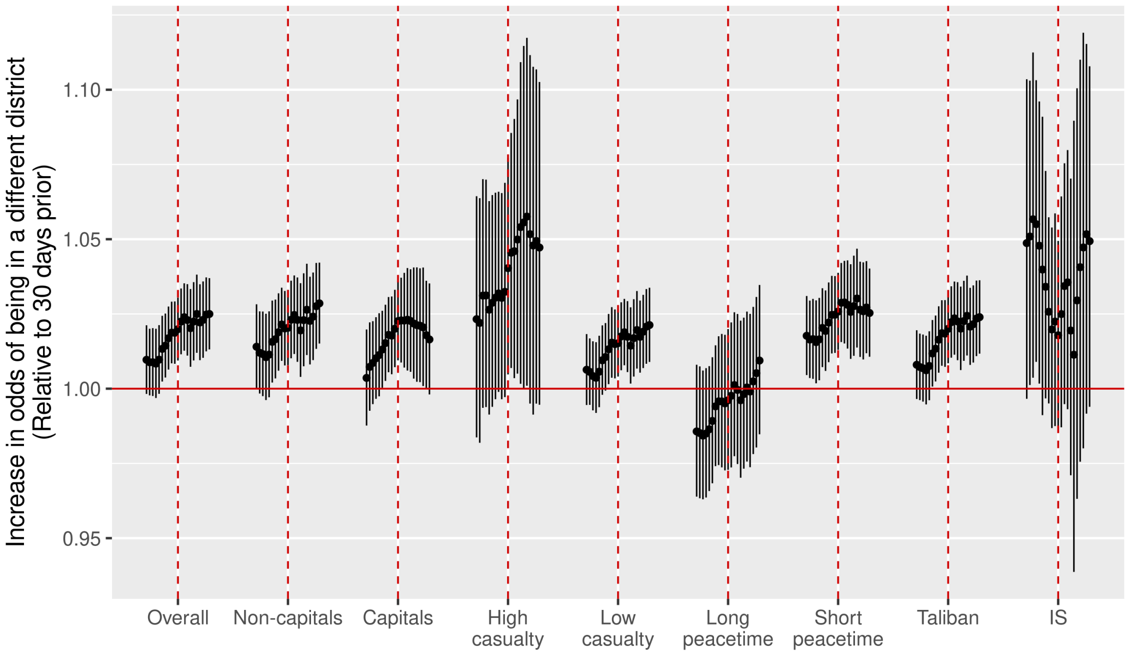

Supplementary Figure 4: Heterogeneous anticipatory effects for different types of violence and affected districts. 30-day displacement results are plotted, for coefficients representing violence happening 10 days prior to 10 days in the future. The dashed red vertical lines represent violence happening on the current day; coefficients to the left correspond to days preceding violence, indicating an anticipatory effect. Exponentiated regression coefficients indicate the increase in the odds that individuals are in a different district than they were 30 days prior. Bars indicate 95% confidence intervals. Estimates are based on 3354 events (overall), 2460 in non-capitals, 894 in non-capitals, 397 high casualty, 2957 low casualty, 1035 long peacetime, 2319 short peacetime, 3134 Taliban and 185 IS.

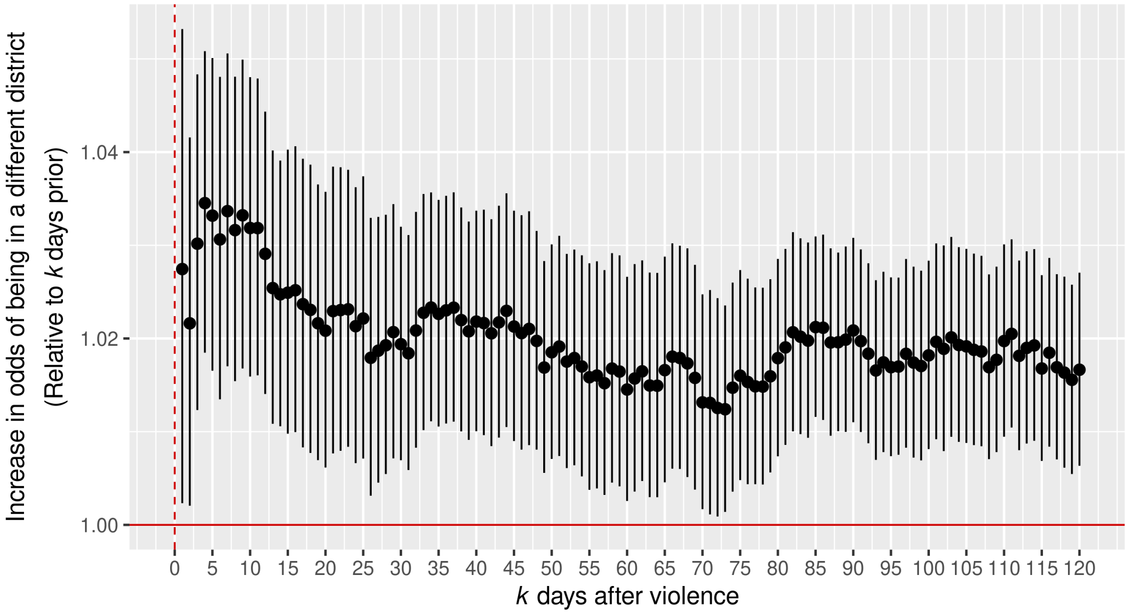

Supplementary Figure 5: Robustness of main results to data restrictions. Figure shows the effect of violence on internal displacement, for those present on the day of the violent event, with analysis restricted to a subset of the data. Specifically, we remove the following observations: (1) Kabul (2) first 60 days and last 90 days of study period (3) days with no "daily modal subscribers" (4) districts with "daily modal subscribers" recorded in fewer than 1095 days (3 years). These restrictions address several potential issues with the data, such as those that might relate to service outages and extreme values. We remove Kabul to investigate if its unique position as the country's capital and most populous district unduly influences results. We find that all qualitative results hold, with only slight changes to the estimated coefficients. Bars indicate 95% confidence intervals. Estimates are based on 2762 violent events.

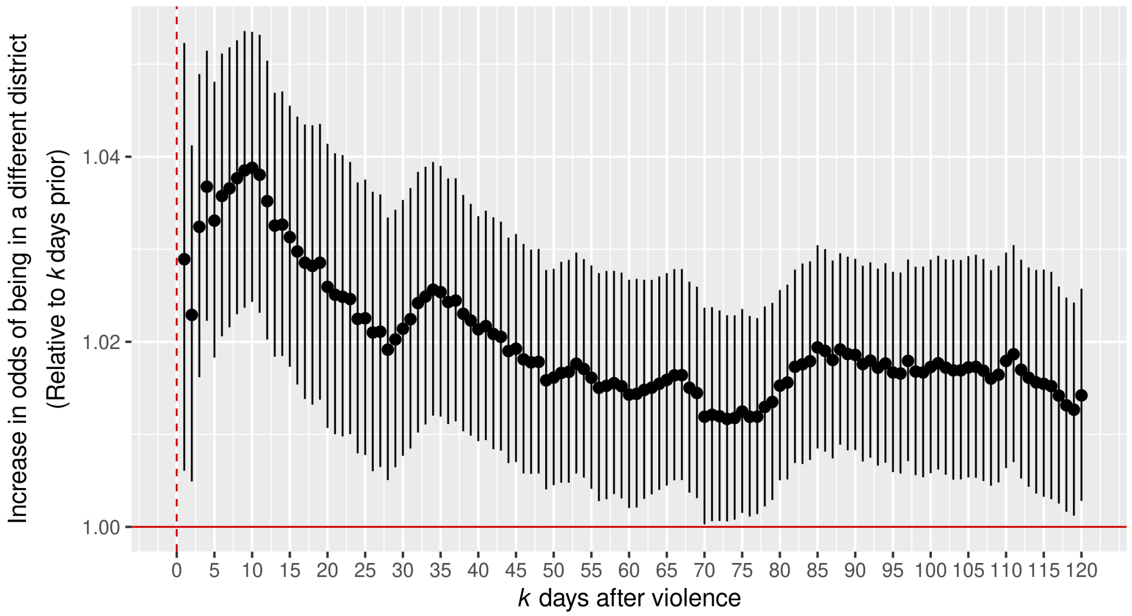

Supplementary Figure 6: Robustness of main results to the addition of time-varying regional control variables. Figure shows the effect of violence on internal displacement, including time-varying controls. Specifically, we include `region*month` as a control, where regions are as described in [3]. This specification helps address the possibility that certain regions might have higher than expected violence and displacement in certain months (for instance, if the fighting season of a region coincided with the regular departure of migrant workers). Results are qualitatively unchanged from our main specification. Bars indicate 95% confidence intervals. Estimates are based on 3354 violent events.

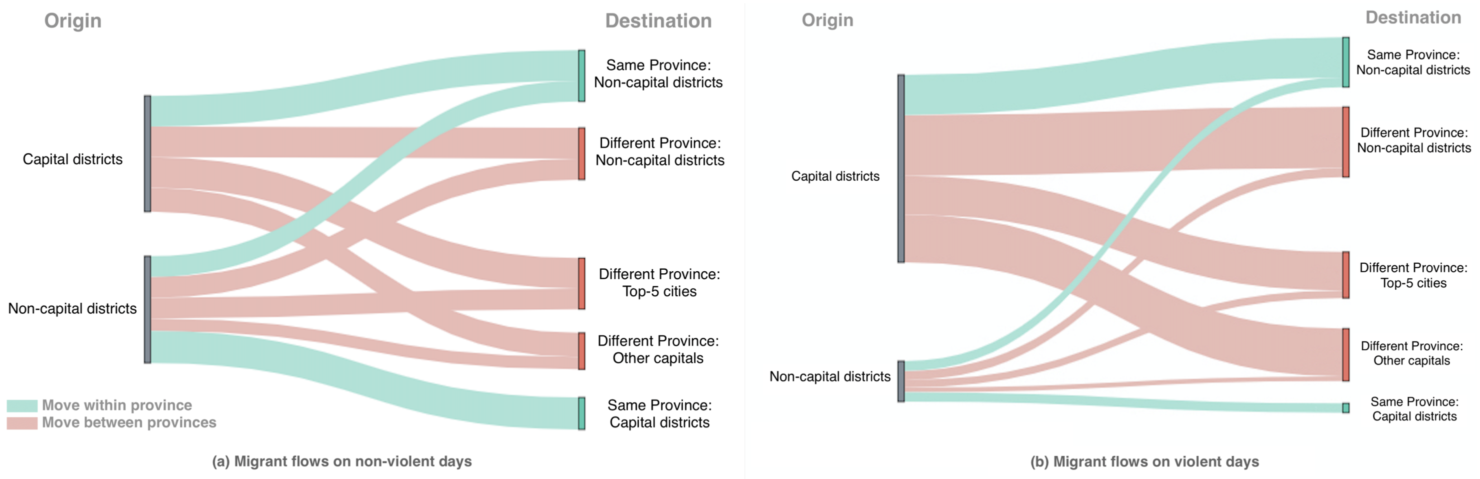

Supplementary Figure 7: Migrant flows on days (a) without violence and (b) with violence. Diagram indicates the proportion of individuals moving between locations of different types, where a move is defined as a change of home district over a 30-day period. For days in which violence occurred (b), the number of individuals moving from capital districts is much larger than from non-capital districts, since capital districts, particularly Kabul, both experience more violent days and have larger population sizes.

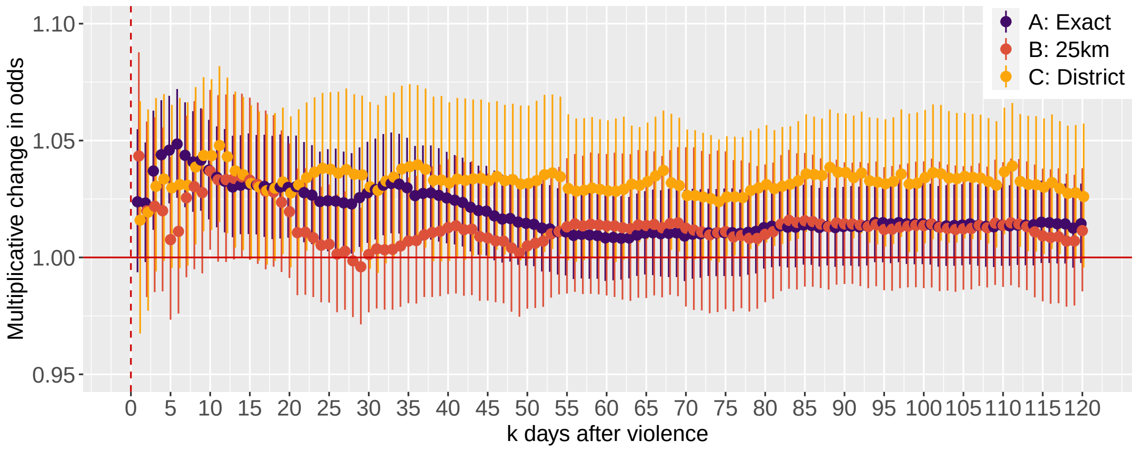

Supplementary Figure 8: Potential implications of missing violence data. Figure shows the effect of violence on internal displacement, separately for violent events recorded with three different levels of spatial resolutions: (A) Events in which the exact location of the event is known and coded (N=1698), (B) Events which occurred within a 25 km radius around a known point (N=789) and (C) Events in which only the district is known (N=969). There are differences in the point estimates, but the confidence intervals of the estimated coefficients for the three types of events overlap. The magnitudes and overall conclusions that we draw in our main analysis are not substantively changed by analyzing these separate groups of events. Bars indicate 95% confidence intervals.

Supplementary Tables

Supplementary Table 1: Can violence be predicted?

	(1)	(2)	(3)	(4)	(5)
Panel A: Model performance					
Linear model R^2	0.000031	0.000029	0.00024	0.00022	0.00028
Random forest R^2	0.00000014	0.0000099	0.000081	0.000020	0.000019
Panel B: Variables used to predict residual violence					
Lags of migration (lags 1-15)	X				X
Trends of migration		X			X
Lags of #subscribers (31-45 days ago)			X		X
Trends of #subscribers				X	X

Notes: Table indicates R^2 of 10 different models that attempt to predict residual violence, where residual violence is defined as the residuals obtained by regressing violence on the control variables in our main Equation (1). Specifically, we first regress the occurrence of violence on day t on the control variables in (1), i.e., $g(\mathbb{E}(T_{dt}|\gamma_d, \lambda_t, T_{d,t+\tau})) = \gamma_d + \lambda_t + \sum_{\tau\in[-30,180]\setminus\{0\}} \beta_\tau T_{d,t+\tau}$, and obtain the residuals $T_{dt} - \hat{\mathbb{E}}(T_{dt})$. We then attempt to predict those residuals using the variables listed in Panel B, where 'lags of migration' refers to 1-15 day lags of the 30-day migration outcome $Y_{dt,30}$, and 'lags of number of subscribers' refers to 1-15 day lags of the number of subscribers observed to be in district d at time $t-30$, i.e., 31-45 days ago. Trends refer to using differences of the migration outcome and number of subscribers as predictors: i.e., $\Delta x_{t-1} = x_{t-1} - x_{t-2}$, $\Delta x_{t-2} = x_{t-2} - x_{t-3}$, etc. Each column of Panel A reports the performance (R^2) of two different models: a linear regression (first row) and a 10-fold cross-validated random forest (second row).

Supplementary Table 2: Full model results for Figure 2a.

Days after violence	Estimate	CI lower	CI upper	p-value
1	1.0276	1.0048	1.0508	0.0171
2	1.0227	1.0047	1.0411	0.0132
3	1.0327	1.0165	1.0492	0.0001
4	1.0377	1.0233	1.0524	0.0000
5	1.0340	1.0191	1.0491	0.0000
6	1.0369	1.0215	1.0525	0.0000
7	1.0380	1.0229	1.0533	0.0000
8	1.0395	1.0248	1.0543	0.0000
9	1.0404	1.0256	1.0554	0.0000
10	1.0403	1.0259	1.0548	0.0000
11	1.0394	1.0247	1.0542	0.0000
12	1.0369	1.0221	1.0519	0.0000
13	1.0341	1.0201	1.0484	0.0000
14	1.0338	1.0198	1.0480	0.0000
15	1.0325	1.0187	1.0465	0.0000
16	1.0311	1.0170	1.0454	0.0000
17	1.0291	1.0147	1.0438	0.0001
18	1.0288	1.0142	1.0437	0.0001
19	1.0291	1.0146	1.0437	0.0001
20	1.0267	1.0120	1.0417	0.0004
21	1.0257	1.0112	1.0405	0.0005
22	1.0253	1.0107	1.0400	0.0006
23	1.0249	1.0107	1.0392	0.0006
24	1.0228	1.0087	1.0371	0.0014
25	1.0231	1.0087	1.0376	0.0016
26	1.0212	1.0065	1.0362	0.0045
27	1.0213	1.0069	1.0359	0.0036
28	1.0196	1.0056	1.0337	0.0058
29	1.0202	1.0065	1.0341	0.0037
30	1.0212	1.0075	1.0351	0.0024

Continued on next page

Supplementary Table 2 – continued from previous page

Days after violence	Estimate	CI lower	CI upper	p-value
31	1.0218	1.0078	1.0359	0.0022
32	1.0237	1.0098	1.0379	0.0008
33	1.0249	1.0110	1.0389	0.0004
34	1.0261	1.0125	1.0399	0.0002
35	1.0262	1.0127	1.0400	0.0001
36	1.0254	1.0120	1.0389	0.0002
37	1.0258	1.0127	1.0391	0.0001
38	1.0246	1.0118	1.0377	0.0002
39	1.0244	1.0117	1.0372	0.0002
40	1.0239	1.0115	1.0363	0.0001
41	1.0240	1.0115	1.0368	0.0002
42	1.0229	1.0102	1.0357	0.0004
43	1.0223	1.0098	1.0350	0.0005
44	1.0209	1.0085	1.0336	0.0010
45	1.0205	1.0079	1.0333	0.0014
46	1.0196	1.0070	1.0325	0.0023
47	1.0192	1.0069	1.0317	0.0022
48	1.0188	1.0065	1.0313	0.0027
49	1.0168	1.0048	1.0289	0.0061
50	1.0171	1.0052	1.0290	0.0045
51	1.0172	1.0052	1.0293	0.0048
52	1.0169	1.0049	1.0291	0.0058
53	1.0177	1.0058	1.0297	0.0034
54	1.0167	1.0049	1.0287	0.0055
55	1.0160	1.0040	1.0281	0.0088
56	1.0152	1.0030	1.0276	0.0144
57	1.0154	1.0032	1.0277	0.0133
58	1.0154	1.0035	1.0275	0.0112
59	1.0151	1.0031	1.0273	0.0133
60	1.0142	1.0021	1.0264	0.0213
61	1.0141	1.0020	1.0264	0.0226

Continued on next page

Supplementary Table 2 – continued from previous page

Days after violence	Estimate	CI lower	CI upper	p-value
62	1.0140	1.0023	1.0258	0.0193
63	1.0147	1.0033	1.0262	0.0114
64	1.0156	1.0043	1.0269	0.0066
65	1.0162	1.0049	1.0275	0.0046
66	1.0169	1.0057	1.0281	0.0029
67	1.0168	1.0056	1.0282	0.0033
68	1.0156	1.0042	1.0272	0.0074
69	1.0152	1.0037	1.0268	0.0092
70	1.0127	1.0009	1.0246	0.0355
71	1.0130	1.0013	1.0248	0.0291
72	1.0128	1.0014	1.0242	0.0273
73	1.0126	1.0014	1.0239	0.0271
74	1.0124	1.0013	1.0235	0.0279
75	1.0132	1.0022	1.0243	0.0181
76	1.0129	1.0020	1.0239	0.0201
77	1.0129	1.0022	1.0237	0.0181
78	1.0138	1.0028	1.0249	0.0138
79	1.0143	1.0035	1.0253	0.0095
80	1.0161	1.0056	1.0267	0.0025
81	1.0161	1.0055	1.0269	0.0028
82	1.0175	1.0069	1.0281	0.0011
83	1.0178	1.0070	1.0287	0.0012
84	1.0182	1.0075	1.0291	0.0008
85	1.0198	1.0088	1.0308	0.0004
86	1.0196	1.0087	1.0306	0.0004
87	1.0188	1.0081	1.0297	0.0006
88	1.0198	1.0094	1.0304	0.0002
89	1.0193	1.0088	1.0299	0.0003
90	1.0194	1.0090	1.0298	0.0002
91	1.0184	1.0078	1.0292	0.0006
92	1.0186	1.0080	1.0293	0.0006

Continued on next page

Supplementary Table 2 – continued from previous page

Days after violence	Estimate	CI lower	CI upper	p-value
93	1.0176	1.0071	1.0282	0.0010
94	1.0185	1.0078	1.0293	0.0006
95	1.0178	1.0070	1.0286	0.0011
96	1.0179	1.0071	1.0288	0.0012
97	1.0192	1.0082	1.0303	0.0006
98	1.0179	1.0066	1.0294	0.0019
99	1.0180	1.0066	1.0296	0.0019
100	1.0187	1.0071	1.0304	0.0016
101	1.0193	1.0076	1.0311	0.0012
102	1.0185	1.0067	1.0304	0.0020
103	1.0183	1.0064	1.0304	0.0024
104	1.0182	1.0063	1.0302	0.0026
105	1.0187	1.0068	1.0308	0.0020
106	1.0188	1.0067	1.0311	0.0022
107	1.0186	1.0064	1.0308	0.0026
108	1.0179	1.0060	1.0299	0.0031
109	1.0180	1.0061	1.0301	0.0030
110	1.0200	1.0080	1.0321	0.0010
111	1.0209	1.0089	1.0331	0.0006
112	1.0190	1.0069	1.0312	0.0020
113	1.0183	1.0062	1.0306	0.0030
114	1.0177	1.0054	1.0303	0.0049
115	1.0175	1.0049	1.0303	0.0064
116	1.0178	1.0052	1.0306	0.0056
117	1.0169	1.0047	1.0291	0.0064
118	1.0159	1.0038	1.0281	0.0098
119	1.0151	1.0030	1.0273	0.0139
120	1.0166	1.0046	1.0287	0.0067

Supplementary Table 3: Full model results for Figure 2b.

Lag	Estimate	CI lower	CI upper	p-value
-30	0.9999	0.9866	1.0135	0.9913
-29	1.0010	0.9878	1.0143	0.8873
-28	1.0016	0.9887	1.0146	0.8125
-27	1.0015	0.9888	1.0144	0.8212
-26	1.0021	0.9905	1.0139	0.7217
-25	1.0008	0.9896	1.0120	0.8944
-24	0.9991	0.9880	1.0104	0.8816
-23	0.9997	0.9884	1.0111	0.9608
-22	0.9968	0.9854	1.0084	0.5869
-21	0.9968	0.9856	1.0082	0.5801
-20	0.9968	0.9851	1.0087	0.5962
-19	0.9977	0.9857	1.0097	0.7017
-18	0.9961	0.9842	1.0081	0.5226
-17	0.9977	0.9859	1.0097	0.7115
-16	0.9995	0.9880	1.0111	0.9327
-15	1.0035	0.9927	1.0144	0.5255
-14	1.0051	0.9951	1.0152	0.3164
-13	1.0063	0.9962	1.0166	0.2212
-12	1.0047	0.9934	1.0161	0.4193
-11	1.0054	0.9939	1.0171	0.3573
-10	1.0097	0.9982	1.0212	0.0975
-9	1.0088	0.9978	1.0200	0.1183
-8	1.0088	0.9975	1.0203	0.1254
-7	1.0084	0.9969	1.0200	0.1538
-6	1.0097	0.9983	1.0213	0.0961
-5	1.0133	1.0024	1.0244	0.0168
-4	1.0144	1.0037	1.0251	0.0080
-3	1.0169	1.0064	1.0275	0.0016
-2	1.0187	1.0085	1.0291	0.0003
-1	1.0187	1.0083	1.0292	0.0004

Continued on next page

Supplementary Table 3 – continued from previous page

Lag	Estimate	CI lower	CI upper	p-value
0	1.0199	1.0094	1.0305	0.0002
1	1.0225	1.0115	1.0335	0.0001
2	1.0239	1.0127	1.0352	0.0000
3	1.0229	1.0111	1.0350	0.0001
4	1.0202	1.0073	1.0333	0.0020
5	1.0225	1.0094	1.0356	0.0007
6	1.0250	1.0120	1.0381	0.0001
7	1.0222	1.0095	1.0349	0.0005
8	1.0230	1.0103	1.0359	0.0004
9	1.0248	1.0125	1.0372	0.0001
10	1.0250	1.0131	1.0370	0.0000
11	1.0262	1.0145	1.0380	0.0000
12	1.0266	1.0149	1.0384	0.0000
13	1.0253	1.0144	1.0362	0.0000
14	1.0256	1.0146	1.0366	0.0000
15	1.0265	1.0158	1.0373	0.0000
16	1.0274	1.0163	1.0386	0.0000
17	1.0256	1.0140	1.0374	0.0000
18	1.0249	1.0128	1.0372	0.0001
19	1.0220	1.0095	1.0346	0.0005
20	1.0202	1.0081	1.0325	0.0010
21	1.0219	1.0097	1.0344	0.0004
22	1.0246	1.0119	1.0374	0.0001
23	1.0246	1.0128	1.0366	0.0000
24	1.0242	1.0123	1.0363	0.0001
25	1.0238	1.0118	1.0359	0.0001
26	1.0232	1.0107	1.0359	0.0003
27	1.0223	1.0091	1.0357	0.0009
28	1.0206	1.0073	1.0340	0.0024
29	1.0214	1.0078	1.0352	0.0020
30	1.0212	1.0075	1.0351	0.0024

Continued on next page

Supplementary Table 3 – continued from previous page

Lag	Estimate	CI lower	CI upper	p-value
31	1.0204	1.0067	1.0343	0.0035
32	1.0203	1.0065	1.0344	0.0039
33	1.0171	1.0033	1.0311	0.0152
34	1.0174	1.0033	1.0316	0.0154
35	1.0148	1.0014	1.0283	0.0301
36	1.0136	1.0004	1.0271	0.0441
37	1.0135	1.0004	1.0268	0.0433
38	1.0135	1.0005	1.0267	0.0422
39	1.0132	1.0003	1.0263	0.0447
40	1.0158	1.0038	1.0279	0.0095
41	1.0157	1.0040	1.0276	0.0086
42	1.0161	1.0039	1.0283	0.0092
43	1.0186	1.0065	1.0309	0.0025
44	1.0193	1.0075	1.0313	0.0014
45	1.0175	1.0055	1.0296	0.0040
46	1.0152	1.0037	1.0268	0.0092
47	1.0140	1.0022	1.0259	0.0201
48	1.0120	1.0000	1.0241	0.0496
49	1.0119	0.9999	1.0239	0.0512
50	1.0117	1.0002	1.0233	0.0458
51	1.0140	1.0020	1.0262	0.0222
52	1.0155	1.0046	1.0265	0.0051
53	1.0146	1.0032	1.0261	0.0121
54	1.0159	1.0049	1.0270	0.0045
55	1.0149	1.0039	1.0260	0.0079
56	1.0104	0.9989	1.0222	0.0771
57	1.0098	0.9979	1.0219	0.1076
58	1.0128	1.0006	1.0252	0.0401
59	1.0121	0.9994	1.0249	0.0627
60	1.0100	0.9977	1.0223	0.1105
61	1.0102	0.9980	1.0226	0.1006

Continued on next page

Supplementary Table 3 – continued from previous page

Lag	Estimate	CI lower	CI upper	p-value
62	1.0111	0.9988	1.0234	0.0763
63	1.0096	0.9977	1.0218	0.1153
64	1.0101	0.9980	1.0223	0.1025
65	1.0109	0.9993	1.0226	0.0645
66	1.0116	1.0005	1.0228	0.0406
67	1.0127	1.0019	1.0236	0.0216
68	1.0118	1.0013	1.0225	0.0279
69	1.0121	1.0014	1.0228	0.0264
70	1.0096	0.9987	1.0206	0.0847
71	1.0100	0.9989	1.0213	0.0788
72	1.0098	0.9986	1.0212	0.0878
73	1.0063	0.9961	1.0167	0.2249
74	1.0055	0.9950	1.0160	0.3063
75	1.0066	0.9959	1.0173	0.2276
76	1.0064	0.9954	1.0176	0.2546
77	1.0054	0.9942	1.0167	0.3453
78	1.0057	0.9951	1.0165	0.2922
79	1.0059	0.9955	1.0165	0.2671
80	1.0069	0.9959	1.0180	0.2179
81	1.0055	0.9942	1.0170	0.3415
82	1.0048	0.9939	1.0158	0.3903
83	1.0025	0.9918	1.0133	0.6517
84	1.0016	0.9908	1.0127	0.7677
85	1.0017	0.9910	1.0126	0.7523
86	1.0029	0.9921	1.0138	0.6043
87	1.0044	0.9936	1.0153	0.4267
88	1.0039	0.9927	1.0151	0.4993
89	1.0026	0.9917	1.0136	0.6400
90	1.0009	0.9888	1.0131	0.8868
91	1.0028	0.9902	1.0156	0.6656
92	1.0004	0.9865	1.0145	0.9523

Continued on next page

Supplementary Table 3 – continued from previous page

Lag	Estimate	CI lower	CI upper	p-value
93	1.0021	0.9885	1.0159	0.7632
94	1.0034	0.9899	1.0171	0.6266
95	1.0035	0.9896	1.0177	0.6217
96	1.0035	0.9897	1.0175	0.6190
97	1.0039	0.9898	1.0182	0.5859
98	1.0016	0.9869	1.0166	0.8292
99	1.0034	0.9886	1.0185	0.6525
100	1.0024	0.9879	1.0171	0.7479
101	1.0012	0.9869	1.0156	0.8734
102	1.0019	0.9877	1.0162	0.7992
103	1.0014	0.9872	1.0157	0.8483
104	0.9994	0.9843	1.0148	0.9405
105	0.9998	0.9847	1.0152	0.9814
106	1.0023	0.9883	1.0166	0.7472
107	1.0039	0.9897	1.0183	0.5939
108	1.0054	0.9915	1.0195	0.4483
109	1.0045	0.9905	1.0187	0.5314
110	1.0035	0.9896	1.0175	0.6245
111	1.0013	0.9885	1.0143	0.8410
112	1.0008	0.9883	1.0135	0.8976
113	1.0025	0.9903	1.0148	0.6909
114	1.0045	0.9926	1.0166	0.4579
115	1.0040	0.9919	1.0161	0.5192
116	1.0041	0.9920	1.0163	0.5064
117	1.0056	0.9936	1.0178	0.3605
118	1.0047	0.9930	1.0166	0.4285
119	1.0037	0.9917	1.0159	0.5485
120	1.0077	0.9958	1.0198	0.2040
121	1.0072	0.9956	1.0190	0.2224
122	1.0055	0.9939	1.0172	0.3563
123	1.0077	0.9955	1.0200	0.2190

Continued on next page

Supplementary Table 3 – continued from previous page

Lag	Estimate	CI lower	CI upper	p-value
124	1.0071	0.9955	1.0189	0.2288
125	1.0086	0.9976	1.0197	0.1254
126	1.0087	0.9979	1.0196	0.1133
127	1.0079	0.9974	1.0185	0.1408
128	1.0068	0.9968	1.0170	0.1831
129	1.0075	0.9976	1.0176	0.1370
130	1.0060	0.9957	1.0164	0.2574
131	1.0085	0.9985	1.0187	0.0969
132	1.0093	0.9989	1.0198	0.0790
133	1.0121	1.0016	1.0228	0.0243
134	1.0113	1.0014	1.0213	0.0249
135	1.0097	0.9999	1.0197	0.0533
136	1.0105	1.0007	1.0204	0.0359
137	1.0088	0.9988	1.0190	0.0850
138	1.0091	0.9988	1.0194	0.0822
139	1.0059	0.9950	1.0170	0.2901
140	1.0046	0.9935	1.0159	0.4204
141	1.0077	0.9965	1.0191	0.1785
142	1.0041	0.9925	1.0158	0.4942
143	1.0035	0.9912	1.0159	0.5787
144	1.0047	0.9923	1.0173	0.4562
145	1.0039	0.9912	1.0167	0.5509
146	1.0031	0.9898	1.0166	0.6450
147	1.0024	0.9889	1.0160	0.7297
148	1.0004	0.9868	1.0142	0.9539
149	1.0015	0.9886	1.0146	0.8217
150	0.9993	0.9851	1.0137	0.9229
151	1.0008	0.9865	1.0153	0.9120
152	1.0020	0.9876	1.0167	0.7843
153	1.0008	0.9866	1.0153	0.9081
154	0.9985	0.9840	1.0131	0.8378

Continued on next page

Supplementary Table 3 – continued from previous page

Lag	Estimate	CI lower	CI upper	p-value
155	1.0005	0.9864	1.0149	0.9424
156	1.0022	0.9882	1.0165	0.7577
157	1.0055	0.9925	1.0187	0.4052
158	1.0042	0.9919	1.0167	0.5029
159	1.0012	0.9885	1.0140	0.8584
160	0.9996	0.9871	1.0123	0.9502
161	1.0017	0.9900	1.0136	0.7720
162	1.0053	0.9936	1.0172	0.3745
163	1.0059	0.9944	1.0175	0.3149
164	1.0063	0.9951	1.0178	0.2721
165	1.0084	0.9970	1.0200	0.1495
166	1.0106	0.9996	1.0217	0.0582
167	1.0120	1.0009	1.0233	0.0348
168	1.0101	0.9984	1.0219	0.0903
169	1.0108	0.9987	1.0230	0.0806
170	1.0106	0.9981	1.0233	0.0974
171	1.0077	0.9953	1.0203	0.2219
172	1.0054	0.9931	1.0179	0.3931
173	1.0049	0.9925	1.0174	0.4388
174	1.0027	0.9905	1.0150	0.6668
175	1.0010	0.9886	1.0135	0.8806
176	0.9983	0.9860	1.0106	0.7812
177	0.9994	0.9874	1.0115	0.9217
178	0.9990	0.9871	1.0111	0.8720
179	1.0015	0.9887	1.0145	0.8169
180	1.0024	0.9889	1.0161	0.7253

Supplementary Table 4: Full model results for Figure 3a.

Days after violence	Type of violence	Estimate	CI lower	CI upper	p-value
1	IS	1.1272	1.0574	1.2015	0.0002
1	Taliban	1.0192	0.9957	1.0433	0.1107
2	IS	1.1167	1.0440	1.1944	0.0013
2	Taliban	1.0158	0.9969	1.0351	0.1010
3	IS	1.1064	1.0522	1.1634	0.0001
3	Taliban	1.0270	1.0097	1.0445	0.0021
4	IS	1.0842	1.0195	1.1531	0.0100
4	Taliban	1.0335	1.0181	1.0490	0.0000
5	IS	1.0653	1.0151	1.1180	0.0102
5	Taliban	1.0310	1.0153	1.0470	0.0001
6	IS	1.0569	1.0054	1.1110	0.0299
6	Taliban	1.0353	1.0191	1.0518	0.0000
7	IS	1.0765	1.0199	1.1362	0.0074
7	Taliban	1.0355	1.0195	1.0517	0.0000
8	IS	1.0890	1.0394	1.1409	0.0003
8	Taliban	1.0364	1.0210	1.0521	0.0000
9	IS	1.0907	1.0401	1.1437	0.0003
9	Taliban	1.0377	1.0222	1.0535	0.0000
10	IS	1.0791	1.0324	1.1278	0.0007
10	Taliban	1.0383	1.0233	1.0537	0.0000
11	IS	1.0710	1.0233	1.1208	0.0032
11	Taliban	1.0380	1.0225	1.0537	0.0000
12	IS	1.0680	1.0201	1.1180	0.0049
12	Taliban	1.0357	1.0203	1.0513	0.0000
13	IS	1.0552	1.0144	1.0976	0.0075
13	Taliban	1.0333	1.0185	1.0483	0.0000
14	IS	1.0561	1.0103	1.1039	0.0159
14	Taliban	1.0334	1.0187	1.0484	0.0000
15	IS	1.0562	1.0139	1.1003	0.0088
15	Taliban	1.0317	1.0172	1.0463	0.0000
16	IS	1.0599	1.0177	1.1038	0.0050

Continued on next page

Supplementary Table 4 – continued from previous page

Days after violence	Type of violence	Estimate	CI lower	CI upper	p-value
16	Taliban	1.0301	1.0154	1.0451	0.0001
17	IS	1.0353	0.9950	1.0771	0.0868
17	Taliban	1.0294	1.0141	1.0450	0.0002
18	IS	1.0351	0.9939	1.0781	0.0959
18	Taliban	1.0288	1.0133	1.0446	0.0002
19	IS	1.0258	0.9772	1.0769	0.3036
19	Taliban	1.0297	1.0144	1.0453	0.0001
20	IS	1.0217	0.9704	1.0756	0.4148
20	Taliban	1.0272	1.0116	1.0430	0.0006
21	IS	1.0256	0.9660	1.0888	0.4083
21	Taliban	1.0261	1.0107	1.0416	0.0008
22	IS	1.0271	0.9663	1.0918	0.3907
22	Taliban	1.0256	1.0103	1.0411	0.0010
23	IS	1.0237	0.9589	1.0929	0.4823
23	Taliban	1.0253	1.0103	1.0405	0.0009
24	IS	1.0234	0.9638	1.0867	0.4507
24	Taliban	1.0234	1.0085	1.0385	0.0020
25	IS	1.0025	0.9542	1.0533	0.9207
25	Taliban	1.0246	1.0094	1.0400	0.0014
26	IS	0.9802	0.9166	1.0482	0.5597
26	Taliban	1.0238	1.0085	1.0394	0.0022
27	IS	0.9839	0.9189	1.0534	0.6410
27	Taliban	1.0241	1.0091	1.0393	0.0016
28	IS	0.9845	0.9200	1.0536	0.6522
28	Taliban	1.0221	1.0076	1.0368	0.0026
29	IS	0.9879	0.9312	1.0480	0.6861
29	Taliban	1.0227	1.0084	1.0373	0.0018
30	IS	0.9958	0.9401	1.0548	0.8867
30	Taliban	1.0231	1.0087	1.0378	0.0017
31	IS	1.0060	0.9539	1.0610	0.8249
31	Taliban	1.0233	1.0085	1.0384	0.0020
32	IS	1.0157	0.9631	1.0710	0.5664

Continued on next page

Supplementary Table 4 – continued from previous page

Days after violence	Type of violence	Estimate	CI lower	CI upper	p-value
32	Taliban	1.0249	1.0100	1.0399	0.0010
33	IS	1.0152	0.9614	1.0722	0.5866
33	Taliban	1.0260	1.0112	1.0409	0.0005
34	IS	1.0185	0.9647	1.0752	0.5081
34	Taliban	1.0271	1.0126	1.0417	0.0002
35	IS	1.0260	0.9729	1.0820	0.3435
35	Taliban	1.0268	1.0125	1.0414	0.0002
36	IS	1.0339	0.9840	1.0864	0.1869
36	Taliban	1.0256	1.0113	1.0401	0.0004
37	IS	1.0299	0.9793	1.0831	0.2525
37	Taliban	1.0261	1.0120	1.0404	0.0003
38	IS	1.0168	0.9418	1.0978	0.6699
38	Taliban	1.0257	1.0119	1.0396	0.0002
39	IS	1.0204	0.9445	1.1024	0.6084
39	Taliban	1.0253	1.0116	1.0391	0.0003
40	IS	1.0197	0.9456	1.0996	0.6130
40	Taliban	1.0248	1.0114	1.0383	0.0003
41	IS	1.0168	0.9471	1.0915	0.6460
41	Taliban	1.0251	1.0114	1.0389	0.0003
42	IS	1.0130	0.9483	1.0821	0.7023
42	Taliban	1.0239	1.0102	1.0378	0.0006
43	IS	1.0118	0.9603	1.0660	0.6596
43	Taliban	1.0232	1.0095	1.0372	0.0009
44	IS	1.0188	0.9747	1.0648	0.4095
44	Taliban	1.0213	1.0075	1.0353	0.0024
45	IS	1.0221	0.9797	1.0664	0.3115
45	Taliban	1.0205	1.0065	1.0347	0.0040
46	IS	1.0193	0.9743	1.0663	0.4066
46	Taliban	1.0199	1.0059	1.0340	0.0052
47	IS	1.0227	0.9751	1.0725	0.3559
47	Taliban	1.0194	1.0057	1.0332	0.0052
48	IS	1.0352	0.9938	1.0783	0.0967

Continued on next page

Supplementary Table 4 – continued from previous page

Days after violence	Type of violence	Estimate	CI lower	CI upper	p-value
48	Taliban	1.0184	1.0047	1.0323	0.0083
49	IS	1.0404	1.0007	1.0816	0.0462
49	Taliban	1.0161	1.0028	1.0297	0.0178
50	IS	1.0352	0.9942	1.0779	0.0937
50	Taliban	1.0166	1.0035	1.0299	0.0127
51	IS	1.0430	0.9993	1.0886	0.0541
51	Taliban	1.0163	1.0031	1.0298	0.0158
52	IS	1.0442	0.9992	1.0911	0.0544
52	Taliban	1.0160	1.0028	1.0295	0.0174
53	IS	1.0471	1.0015	1.0948	0.0429
53	Taliban	1.0171	1.0041	1.0303	0.0099
54	IS	1.0446	0.9932	1.0987	0.0904
54	Taliban	1.0160	1.0031	1.0292	0.0151
55	IS	1.0488	0.9890	1.1122	0.1114
55	Taliban	1.0151	1.0022	1.0282	0.0220
56	IS	1.0606	1.0026	1.1219	0.0402
56	Taliban	1.0139	1.0007	1.0272	0.0384
57	IS	1.0559	1.0022	1.1124	0.0413
57	Taliban	1.0142	1.0010	1.0275	0.0343
58	IS	1.0521	1.0004	1.1065	0.0482
58	Taliban	1.0145	1.0016	1.0276	0.0279
59	IS	1.0493	1.0032	1.0975	0.0356
59	Taliban	1.0143	1.0012	1.0275	0.0318
60	IS	1.0394	0.9960	1.0846	0.0759
60	Taliban	1.0135	1.0004	1.0268	0.0430
61	IS	1.0423	0.9990	1.0874	0.0556
61	Taliban	1.0135	1.0003	1.0269	0.0457
62	IS	1.0453	1.0029	1.0894	0.0358
62	Taliban	1.0135	1.0007	1.0264	0.0388
63	IS	1.0394	0.9976	1.0828	0.0647
63	Taliban	1.0145	1.0021	1.0270	0.0214
64	IS	1.0396	0.9953	1.0858	0.0805

Continued on next page

Supplementary Table 4 – continued from previous page

Days after violence	Type of violence	Estimate	CI lower	CI upper	p-value
64	Taliban	1.0153	1.0032	1.0276	0.0130
65	IS	1.0459	1.0019	1.0918	0.0408
65	Taliban	1.0157	1.0037	1.0279	0.0104
66	IS	1.0385	0.9942	1.0848	0.0891
66	Taliban	1.0167	1.0047	1.0289	0.0061
67	IS	1.0324	0.9873	1.0795	0.1620
67	Taliban	1.0168	1.0048	1.0290	0.0061
68	IS	1.0387	0.9937	1.0856	0.0928
68	Taliban	1.0153	1.0030	1.0277	0.0148
69	IS	1.0408	0.9985	1.0849	0.0591
69	Taliban	1.0149	1.0027	1.0273	0.0166
70	IS	1.0163	0.9451	1.0929	0.6624
70	Taliban	1.0133	1.0011	1.0257	0.0329
71	IS	1.0187	0.9532	1.0887	0.5848
71	Taliban	1.0138	1.0017	1.0260	0.0256
72	IS	1.0161	0.9526	1.0839	0.6276
72	Taliban	1.0136	1.0018	1.0257	0.0241
73	IS	1.0146	0.9502	1.0834	0.6645
73	Taliban	1.0139	1.0022	1.0257	0.0197
74	IS	1.0243	0.9629	1.0896	0.4469
74	Taliban	1.0135	1.0020	1.0252	0.0216
75	IS	1.0347	0.9830	1.0891	0.1919
75	Taliban	1.0143	1.0026	1.0260	0.0161
76	IS	1.0324	0.9826	1.0846	0.2060
76	Taliban	1.0140	1.0024	1.0258	0.0183
77	IS	1.0263	0.9760	1.0792	0.3108
77	Taliban	1.0142	1.0027	1.0258	0.0152
78	IS	1.0337	0.9841	1.0857	0.1862
78	Taliban	1.0149	1.0032	1.0267	0.0127
79	IS	1.0305	0.9750	1.0891	0.2873
79	Taliban	1.0153	1.0038	1.0270	0.0093
80	IS	1.0387	0.9909	1.0888	0.1146

Continued on next page

Supplementary Table 4 – continued from previous page

Days after violence	Type of violence	Estimate	CI lower	CI upper	p-value
80	Taliban	1.0168	1.0056	1.0281	0.0032
81	IS	1.0436	0.9896	1.1005	0.1158
81	Taliban	1.0166	1.0053	1.0281	0.0040
82	IS	1.0497	0.9965	1.1057	0.0676
82	Taliban	1.0176	1.0063	1.0291	0.0021
83	IS	1.0576	0.9948	1.1244	0.0731
83	Taliban	1.0177	1.0062	1.0293	0.0025
84	IS	1.0594	0.9975	1.1252	0.0603
84	Taliban	1.0181	1.0066	1.0296	0.0019
85	IS	1.0614	1.0026	1.1237	0.0404
85	Taliban	1.0197	1.0079	1.0317	0.0010
86	IS	1.0616	1.0036	1.1230	0.0371
86	Taliban	1.0197	1.0079	1.0316	0.0011
87	IS	1.0606	1.0053	1.1189	0.0314
87	Taliban	1.0189	1.0073	1.0306	0.0013
88	IS	1.0655	1.0105	1.1236	0.0190
88	Taliban	1.0199	1.0088	1.0312	0.0004
89	IS	1.0650	1.0121	1.1207	0.0154
89	Taliban	1.0192	1.0079	1.0307	0.0008
90	IS	1.0548	1.0054	1.1066	0.0293
90	Taliban	1.0196	1.0084	1.0309	0.0006
91	IS	1.0494	1.0005	1.1007	0.0475
91	Taliban	1.0187	1.0073	1.0302	0.0012
92	IS	1.0543	1.0031	1.1080	0.0373
92	Taliban	1.0186	1.0073	1.0300	0.0012
93	IS	1.0465	0.9998	1.0954	0.0508
93	Taliban	1.0177	1.0065	1.0291	0.0019
94	IS	1.0450	0.9995	1.0926	0.0529
94	Taliban	1.0187	1.0074	1.0302	0.0012
95	IS	1.0493	1.0028	1.0979	0.0375
95	Taliban	1.0179	1.0065	1.0294	0.0020
96	IS	1.0530	1.0079	1.1000	0.0207

Continued on next page

Supplementary Table 4 – continued from previous page

Days after violence	Type of violence	Estimate	CI lower	CI upper	p-value
96	Taliban	1.0180	1.0065	1.0296	0.0020
97	IS	1.0536	1.0096	1.0994	0.0164
97	Taliban	1.0193	1.0076	1.0311	0.0011
98	IS	1.0248	0.9688	1.0840	0.3933
98	Taliban	1.0190	1.0072	1.0310	0.0016
99	IS	1.0150	0.9603	1.0728	0.5994
99	Taliban	1.0194	1.0074	1.0315	0.0014
100	IS	1.0167	0.9623	1.0742	0.5548
100	Taliban	1.0201	1.0080	1.0324	0.0011
101	IS	1.0208	0.9736	1.0703	0.3934
101	Taliban	1.0207	1.0084	1.0332	0.0010
102	IS	1.0147	0.9677	1.0639	0.5469
102	Taliban	1.0201	1.0076	1.0327	0.0016
103	IS	1.0138	0.9717	1.0576	0.5267
103	Taliban	1.0200	1.0071	1.0330	0.0022
104	IS	1.0078	0.9701	1.0471	0.6883
104	Taliban	1.0200	1.0072	1.0331	0.0022
105	IS	1.0010	0.9621	1.0414	0.9619
105	Taliban	1.0208	1.0079	1.0339	0.0015
106	IS	0.9945	0.9528	1.0379	0.7986
106	Taliban	1.0211	1.0080	1.0343	0.0015
107	IS	0.9930	0.9487	1.0393	0.7625
107	Taliban	1.0210	1.0081	1.0342	0.0014
108	IS	0.9965	0.9536	1.0414	0.8766
108	Taliban	1.0201	1.0074	1.0331	0.0019
109	IS	0.9938	0.9490	1.0408	0.7935
109	Taliban	1.0203	1.0074	1.0333	0.0019
110	IS	0.9908	0.9488	1.0346	0.6742
110	Taliban	1.0224	1.0095	1.0354	0.0006
111	IS	0.9902	0.9446	1.0381	0.6842
111	Taliban	1.0233	1.0105	1.0364	0.0003
112	IS	0.9988	0.9599	1.0392	0.9513

Continued on next page

Supplementary Table 4 – continued from previous page

Days after violence	Type of violence	Estimate	CI lower	CI upper	p-value
112	Taliban	1.0209	1.0079	1.0340	0.0016
113	IS	1.0015	0.9600	1.0448	0.9439
113	Taliban	1.0201	1.0071	1.0333	0.0024
114	IS	1.0064	0.9675	1.0468	0.7509
114	Taliban	1.0193	1.0059	1.0328	0.0046
115	IS	1.0124	0.9756	1.0506	0.5148
115	Taliban	1.0190	1.0054	1.0328	0.0060
116	IS	1.0204	0.9878	1.0541	0.2220
116	Taliban	1.0191	1.0054	1.0329	0.0061
117	IS	1.0259	0.9944	1.0583	0.1077
117	Taliban	1.0179	1.0046	1.0313	0.0079
118	IS	1.0251	0.9919	1.0594	0.1404
118	Taliban	1.0167	1.0035	1.0301	0.0133
119	IS	1.0217	0.9869	1.0578	0.2240
119	Taliban	1.0159	1.0027	1.0292	0.0184
120	IS	1.0240	0.9937	1.0553	0.1220
120	Taliban	1.0174	1.0042	1.0308	0.0097

Supplementary Table 5: Full model results for Figure 3b.

Days after violence	Type of violence	Estimate	CI lower	CI upper	p-value
1	>=11 casualties	1.1106	1.0441	1.1815	0.0009
1	<11 casualties	1.0159	0.9919	1.0404	0.1949
2	>=11 casualties	1.0755	1.0134	1.1414	0.0164
2	<11 casualties	1.0151	0.9956	1.0350	0.1304
3	>=11 casualties	1.0856	1.0221	1.1531	0.0076
3	<11 casualties	1.0257	1.0084	1.0434	0.0035
4	>=11 casualties	1.0920	1.0322	1.1552	0.0022
4	<11 casualties	1.0305	1.0154	1.0458	0.0001
5	>=11 casualties	1.0842	1.0233	1.1488	0.0062
5	<11 casualties	1.0271	1.0123	1.0421	0.0003
6	>=11 casualties	1.0930	1.0293	1.1607	0.0037
6	<11 casualties	1.0289	1.0137	1.0443	0.0002
7	>=11 casualties	1.0922	1.0283	1.1600	0.0041
7	<11 casualties	1.0301	1.0156	1.0448	0.0000
8	>=11 casualties	1.0937	1.0343	1.1566	0.0017
8	<11 casualties	1.0315	1.0173	1.0460	0.0000
9	>=11 casualties	1.0799	1.0213	1.1419	0.0069
9	<11 casualties	1.0339	1.0198	1.0483	0.0000
10	>=11 casualties	1.0829	1.0262	1.1428	0.0037
10	<11 casualties	1.0334	1.0193	1.0476	0.0000
11	>=11 casualties	1.0751	1.0209	1.1322	0.0061
11	<11 casualties	1.0330	1.0185	1.0478	0.0000
12	>=11 casualties	1.0731	1.0193	1.1297	0.0071
12	<11 casualties	1.0304	1.0160	1.0450	0.0000
13	>=11 casualties	1.0651	1.0143	1.1185	0.0114
13	<11 casualties	1.0280	1.0141	1.0420	0.0001
14	>=11 casualties	1.0623	1.0128	1.1143	0.0131
14	<11 casualties	1.0280	1.0139	1.0422	0.0001
15	>=11 casualties	1.0639	1.0141	1.1162	0.0113
15	<11 casualties	1.0263	1.0127	1.0401	0.0001
16	>=11 casualties	1.0654	1.0151	1.1182	0.0102

Continued on next page

Supplementary Table 5 – continued from previous page

Days after violence	Type of violence	Estimate	CI lower	CI upper	p-value
16	<11 casualties	1.0246	1.0108	1.0385	0.0004
17	>=11 casualties	1.0585	1.0095	1.1100	0.0187
17	<11 casualties	1.0234	1.0090	1.0380	0.0014
18	>=11 casualties	1.0586	1.0096	1.1099	0.0184
18	<11 casualties	1.0230	1.0085	1.0378	0.0019
19	>=11 casualties	1.0458	0.9975	1.0964	0.0634
19	<11 casualties	1.0244	1.0101	1.0389	0.0008
20	>=11 casualties	1.0435	0.9947	1.0946	0.0815
20	<11 casualties	1.0218	1.0074	1.0364	0.0029
21	>=11 casualties	1.0463	0.9970	1.0980	0.0663
21	<11 casualties	1.0203	1.0062	1.0345	0.0045
22	>=11 casualties	1.0402	0.9925	1.0903	0.0999
22	<11 casualties	1.0204	1.0062	1.0347	0.0048
23	>=11 casualties	1.0396	0.9920	1.0893	0.1041
23	<11 casualties	1.0202	1.0062	1.0343	0.0045
24	>=11 casualties	1.0326	0.9852	1.0823	0.1807
24	<11 casualties	1.0188	1.0049	1.0328	0.0077
25	>=11 casualties	1.0289	0.9815	1.0785	0.2361
25	<11 casualties	1.0195	1.0052	1.0339	0.0072
26	>=11 casualties	1.0255	0.9784	1.0748	0.2933
26	<11 casualties	1.0178	1.0030	1.0328	0.0179
27	>=11 casualties	1.0286	0.9820	1.0774	0.2334
27	<11 casualties	1.0177	1.0031	1.0324	0.0171
28	>=11 casualties	1.0250	0.9800	1.0720	0.2814
28	<11 casualties	1.0163	1.0020	1.0307	0.0249
29	>=11 casualties	1.0248	0.9806	1.0710	0.2759
29	<11 casualties	1.0172	1.0031	1.0316	0.0168
30	>=11 casualties	1.0283	0.9826	1.0761	0.2286
30	<11 casualties	1.0180	1.0036	1.0326	0.0143
31	>=11 casualties	1.0268	0.9803	1.0755	0.2629
31	<11 casualties	1.0188	1.0043	1.0336	0.0111
32	>=11 casualties	1.0308	0.9846	1.0792	0.1950

Continued on next page

Supplementary Table 5 – continued from previous page

Days after violence	Type of violence	Estimate	CI lower	CI upper	p-value
32	<11 casualties	1.0205	1.0058	1.0354	0.0062
33	>=11 casualties	1.0313	0.9840	1.0809	0.1988
33	<11 casualties	1.0218	1.0072	1.0367	0.0034
34	>=11 casualties	1.0366	0.9885	1.0870	0.1383
34	<11 casualties	1.0226	1.0082	1.0373	0.0020
35	>=11 casualties	1.0401	0.9920	1.0906	0.1036
35	<11 casualties	1.0226	1.0077	1.0377	0.0029
36	>=11 casualties	1.0460	0.9973	1.0969	0.0643
36	<11 casualties	1.0211	1.0060	1.0365	0.0060
37	>=11 casualties	1.0474	0.9981	1.0992	0.0597
37	<11 casualties	1.0214	1.0067	1.0363	0.0041
38	>=11 casualties	1.0495	0.9982	1.1034	0.0587
38	<11 casualties	1.0199	1.0054	1.0346	0.0070
39	>=11 casualties	1.0501	0.9988	1.1040	0.0557
39	<11 casualties	1.0193	1.0048	1.0340	0.0090
40	>=11 casualties	1.0521	0.9995	1.1074	0.0524
40	<11 casualties	1.0182	1.0039	1.0326	0.0125
41	>=11 casualties	1.0556	1.0029	1.1111	0.0385
41	<11 casualties	1.0180	1.0035	1.0328	0.0150
42	>=11 casualties	1.0539	1.0007	1.1100	0.0471
42	<11 casualties	1.0169	1.0020	1.0320	0.0257
43	>=11 casualties	1.0469	0.9909	1.1062	0.1024
43	<11 casualties	1.0169	1.0021	1.0320	0.0254
44	>=11 casualties	1.0483	0.9930	1.1068	0.0881
44	<11 casualties	1.0153	1.0005	1.0304	0.0429
45	>=11 casualties	1.0476	0.9935	1.1046	0.0854
45	<11 casualties	1.0148	0.9998	1.0300	0.0527
46	>=11 casualties	1.0478	0.9945	1.1041	0.0798
46	<11 casualties	1.0139	0.9993	1.0287	0.0627
47	>=11 casualties	1.0489	0.9954	1.1053	0.0736
47	<11 casualties	1.0132	0.9993	1.0272	0.0622
48	>=11 casualties	1.0532	0.9997	1.1096	0.0512

Continued on next page

Supplementary Table 5 – continued from previous page

Days after violence	Type of violence	Estimate	CI lower	CI upper	p-value
48	<11 casualties	1.0121	0.9982	1.0261	0.0877
49	>=11 casualties	1.0562	1.0017	1.1136	0.0431
49	<11 casualties	1.0096	0.9962	1.0232	0.1609
50	>=11 casualties	1.0575	1.0031	1.1149	0.0380
50	<11 casualties	1.0098	0.9966	1.0231	0.1455
51	>=11 casualties	1.0621	1.0063	1.1211	0.0288
51	<11 casualties	1.0093	0.9963	1.0226	0.1619
52	>=11 casualties	1.0630	1.0059	1.1233	0.0301
52	<11 casualties	1.0088	0.9957	1.0221	0.1880
53	>=11 casualties	1.0644	1.0073	1.1249	0.0266
53	<11 casualties	1.0094	0.9966	1.0223	0.1517
54	>=11 casualties	1.0626	1.0059	1.1226	0.0300
54	<11 casualties	1.0084	0.9957	1.0213	0.1945
55	>=11 casualties	1.0666	1.0095	1.1270	0.0217
55	<11 casualties	1.0072	0.9944	1.0201	0.2716
56	>=11 casualties	1.0692	1.0110	1.1308	0.0191
56	<11 casualties	1.0061	0.9936	1.0187	0.3406
57	>=11 casualties	1.0671	1.0093	1.1281	0.0223
57	<11 casualties	1.0066	0.9941	1.0192	0.3041
58	>=11 casualties	1.0656	1.0075	1.1271	0.0264
58	<11 casualties	1.0069	0.9945	1.0196	0.2763
59	>=11 casualties	1.0618	1.0052	1.1217	0.0320
59	<11 casualties	1.0071	0.9948	1.0197	0.2596
60	>=11 casualties	1.0589	1.0040	1.1167	0.0350
60	<11 casualties	1.0064	0.9938	1.0191	0.3203
61	>=11 casualties	1.0572	1.0035	1.1139	0.0365
61	<11 casualties	1.0066	0.9939	1.0195	0.3087
62	>=11 casualties	1.0519	0.9991	1.1074	0.0540
62	<11 casualties	1.0071	0.9945	1.0199	0.2712
63	>=11 casualties	1.0468	0.9957	1.1005	0.0733
63	<11 casualties	1.0087	0.9959	1.0215	0.1827
64	>=11 casualties	1.0464	0.9955	1.0999	0.0748

Continued on next page

Supplementary Table 5 – continued from previous page

Days after violence	Type of violence	Estimate	CI lower	CI upper	p-value
64	<11 casualties	1.0097	0.9973	1.0223	0.1264
65	>=11 casualties	1.0465	0.9958	1.0998	0.0730
65	<11 casualties	1.0104	0.9981	1.0229	0.0964
66	>=11 casualties	1.0427	0.9921	1.0958	0.0997
66	<11 casualties	1.0115	0.9996	1.0237	0.0590
67	>=11 casualties	1.0436	0.9925	1.0972	0.0954
67	<11 casualties	1.0115	0.9994	1.0238	0.0631
68	>=11 casualties	1.0418	0.9900	1.0962	0.1155
68	<11 casualties	1.0105	0.9984	1.0227	0.0898
69	>=11 casualties	1.0435	0.9912	1.0986	0.1047
69	<11 casualties	1.0099	0.9978	1.0222	0.1098
70	>=11 casualties	1.0261	0.9724	1.0828	0.3471
70	<11 casualties	1.0090	0.9964	1.0219	0.1622
71	>=11 casualties	1.0265	0.9733	1.0825	0.3357
71	<11 casualties	1.0095	0.9968	1.0223	0.1431
72	>=11 casualties	1.0222	0.9695	1.0778	0.4158
72	<11 casualties	1.0097	0.9970	1.0226	0.1339
73	>=11 casualties	1.0235	0.9718	1.0779	0.3793
73	<11 casualties	1.0093	0.9967	1.0221	0.1472
74	>=11 casualties	1.0234	0.9713	1.0784	0.3858
74	<11 casualties	1.0092	0.9966	1.0220	0.1526
75	>=11 casualties	1.0257	0.9729	1.0812	0.3466
75	<11 casualties	1.0100	0.9976	1.0225	0.1155
76	>=11 casualties	1.0278	0.9755	1.0829	0.3028
76	<11 casualties	1.0093	0.9969	1.0218	0.1415
77	>=11 casualties	1.0309	0.9789	1.0856	0.2497
77	<11 casualties	1.0088	0.9962	1.0215	0.1712
78	>=11 casualties	1.0340	0.9815	1.0893	0.2085
78	<11 casualties	1.0095	0.9969	1.0222	0.1398
79	>=11 casualties	1.0383	0.9840	1.0956	0.1702
79	<11 casualties	1.0094	0.9971	1.0219	0.1356
80	>=11 casualties	1.0444	0.9900	1.1018	0.1114

Continued on next page

Supplementary Table 5 – continued from previous page

Days after violence	Type of violence	Estimate	CI lower	CI upper	p-value
80	<11 casualties	1.0107	0.9983	1.0232	0.0908
81	>=11 casualties	1.0473	0.9919	1.1058	0.0958
81	<11 casualties	1.0105	0.9981	1.0231	0.0976
82	>=11 casualties	1.0499	0.9947	1.1081	0.0774
82	<11 casualties	1.0117	0.9994	1.0242	0.0617
83	>=11 casualties	1.0517	0.9955	1.1110	0.0718
83	<11 casualties	1.0121	0.9998	1.0245	0.0531
84	>=11 casualties	1.0531	0.9977	1.1116	0.0607
84	<11 casualties	1.0124	1.0001	1.0247	0.0474
85	>=11 casualties	1.0548	1.0000	1.1127	0.0500
85	<11 casualties	1.0138	1.0009	1.0269	0.0355
86	>=11 casualties	1.0555	1.0004	1.1136	0.0484
86	<11 casualties	1.0135	1.0005	1.0267	0.0418
87	>=11 casualties	1.0553	1.0007	1.1129	0.0469
87	<11 casualties	1.0126	0.9998	1.0255	0.0532
88	>=11 casualties	1.0555	1.0017	1.1122	0.0429
88	<11 casualties	1.0135	1.0009	1.0263	0.0358
89	>=11 casualties	1.0553	1.0013	1.1122	0.0446
89	<11 casualties	1.0131	1.0004	1.0259	0.0440
90	>=11 casualties	1.0541	1.0025	1.1084	0.0395
90	<11 casualties	1.0131	1.0002	1.0261	0.0470
91	>=11 casualties	1.0526	1.0025	1.1052	0.0392
91	<11 casualties	1.0121	0.9996	1.0247	0.0588
92	>=11 casualties	1.0516	1.0020	1.1036	0.0413
92	<11 casualties	1.0124	1.0001	1.0250	0.0489
93	>=11 casualties	1.0513	1.0024	1.1025	0.0393
93	<11 casualties	1.0113	0.9985	1.0243	0.0833
94	>=11 casualties	1.0534	1.0041	1.1050	0.0332
94	<11 casualties	1.0122	0.9994	1.0252	0.0621
95	>=11 casualties	1.0528	1.0032	1.1049	0.0365
95	<11 casualties	1.0114	0.9987	1.0243	0.0791
96	>=11 casualties	1.0505	1.0015	1.1020	0.0431

Continued on next page

Supplementary Table 5 – continued from previous page

Days after violence	Type of violence	Estimate	CI lower	CI upper	p-value
96	<11 casualties	1.0118	0.9990	1.0248	0.0719
97	>=11 casualties	1.0509	1.0015	1.1026	0.0432
97	<11 casualties	1.0133	1.0003	1.0265	0.0451
98	>=11 casualties	1.0449	1.0000	1.0919	0.0499
98	<11 casualties	1.0126	0.9992	1.0261	0.0648
99	>=11 casualties	1.0441	0.9994	1.0908	0.0533
99	<11 casualties	1.0127	0.9992	1.0264	0.0649
100	>=11 casualties	1.0447	1.0000	1.0914	0.0497
100	<11 casualties	1.0134	1.0001	1.0270	0.0487
101	>=11 casualties	1.0472	1.0030	1.0934	0.0359
101	<11 casualties	1.0137	1.0002	1.0274	0.0473
102	>=11 casualties	1.0511	1.0052	1.0990	0.0287
102	<11 casualties	1.0124	0.9984	1.0265	0.0824
103	>=11 casualties	1.0487	1.0035	1.0960	0.0345
103	<11 casualties	1.0124	0.9980	1.0269	0.0909
104	>=11 casualties	1.0499	1.0050	1.0968	0.0289
104	<11 casualties	1.0120	0.9976	1.0265	0.1019
105	>=11 casualties	1.0476	1.0022	1.0950	0.0395
105	<11 casualties	1.0128	0.9984	1.0275	0.0817
106	>=11 casualties	1.0478	1.0024	1.0954	0.0390
106	<11 casualties	1.0129	0.9984	1.0276	0.0807
107	>=11 casualties	1.0481	1.0028	1.0954	0.0371
107	<11 casualties	1.0126	0.9983	1.0271	0.0853
108	>=11 casualties	1.0454	1.0016	1.0910	0.0418
108	<11 casualties	1.0121	0.9981	1.0263	0.0896
109	>=11 casualties	1.0450	1.0011	1.0908	0.0443
109	<11 casualties	1.0123	0.9981	1.0268	0.0903
110	>=11 casualties	1.0438	1.0000	1.0896	0.0500
110	<11 casualties	1.0147	1.0004	1.0292	0.0434
111	>=11 casualties	1.0463	1.0016	1.0930	0.0423
111	<11 casualties	1.0153	1.0011	1.0297	0.0345
112	>=11 casualties	1.0395	0.9984	1.0823	0.0601

Continued on next page

Supplementary Table 5 – continued from previous page

Days after violence	Type of violence	Estimate	CI lower	CI upper	p-value
112	<11 casualties	1.0139	0.9996	1.0284	0.0566
113	>=11 casualties	1.0404	0.9994	1.0831	0.0536
113	<11 casualties	1.0132	0.9989	1.0276	0.0705
114	>=11 casualties	1.0433	1.0025	1.0857	0.0371
114	<11 casualties	1.0123	0.9977	1.0270	0.0983
115	>=11 casualties	1.0411	0.9999	1.0841	0.0507
115	<11 casualties	1.0124	0.9977	1.0274	0.0996
116	>=11 casualties	1.0389	0.9984	1.0810	0.0601
116	<11 casualties	1.0130	0.9981	1.0280	0.0872
117	>=11 casualties	1.0379	0.9971	1.0804	0.0693
117	<11 casualties	1.0121	0.9978	1.0266	0.0978
118	>=11 casualties	1.0359	0.9951	1.0784	0.0855
118	<11 casualties	1.0112	0.9970	1.0257	0.1226
119	>=11 casualties	1.0359	0.9955	1.0780	0.0826
119	<11 casualties	1.0104	0.9961	1.0249	0.1557
120	>=11 casualties	1.0360	0.9951	1.0787	0.0853
120	<11 casualties	1.0122	0.9978	1.0269	0.0978

Supplementary Table 6: Full model results for Figure 3c.

Days after violence	Type of violence	Estimate	CI lower	CI upper	p-value
1	>=60 days since last violence	0.9992	0.9548	1.0457	0.9732
1	<60 days since last violence	1.0354	1.0109	1.0605	0.0045
2	>=60 days since last violence	1.0032	0.9680	1.0396	0.8620
2	<60 days since last violence	1.0269	1.0041	1.0503	0.0205
3	>=60 days since last violence	1.0135	0.9788	1.0495	0.4506
3	<60 days since last violence	1.0363	1.0166	1.0565	0.0003
4	>=60 days since last violence	1.0158	0.9848	1.0477	0.3206
4	<60 days since last violence	1.0424	1.0243	1.0607	0.0000
5	>=60 days since last violence	1.0005	0.9703	1.0316	0.9754
5	<60 days since last violence	1.0445	1.0260	1.0633	0.0000
6	>=60 days since last violence	1.0063	0.9768	1.0366	0.6801
6	<60 days since last violence	1.0456	1.0259	1.0655	0.0000
7	>=60 days since last violence	1.0016	0.9740	1.0300	0.9099
7	<60 days since last violence	1.0494	1.0299	1.0693	0.0000
8	>=60 days since last violence	1.0093	0.9826	1.0368	0.4973
8	<60 days since last violence	1.0473	1.0287	1.0662	0.0000
9	>=60 days since last violence	1.0118	0.9855	1.0388	0.3827
9	<60 days since last violence	1.0471	1.0287	1.0658	0.0000
10	>=60 days since last violence	1.0120	0.9860	1.0386	0.3689
10	<60 days since last violence	1.0465	1.0287	1.0645	0.0000
11	>=60 days since last violence	1.0099	0.9823	1.0383	0.4850
11	<60 days since last violence	1.0457	1.0279	1.0638	0.0000
12	>=60 days since last violence	1.0071	0.9799	1.0350	0.6140
12	<60 days since last violence	1.0432	1.0252	1.0615	0.0000
13	>=60 days since last violence	1.0077	0.9806	1.0356	0.5800
13	<60 days since last violence	1.0386	1.0219	1.0555	0.0000
14	>=60 days since last violence	1.0087	0.9811	1.0372	0.5400
14	<60 days since last violence	1.0374	1.0208	1.0544	0.0000
15	>=60 days since last violence	1.0087	0.9816	1.0366	0.5332
15	<60 days since last violence	1.0356	1.0189	1.0526	0.0000
16	>=60 days since last violence	1.0066	0.9794	1.0344	0.6394

Continued on next page

Supplementary Table 6 – continued from previous page

Days after violence	Type of violence	Estimate	CI lower	CI upper	p-value
16	<60 days since last violence	1.0344	1.0173	1.0518	0.0001
17	>=60 days since last violence	1.0053	0.9785	1.0329	0.7011
17	<60 days since last violence	1.0321	1.0144	1.0501	0.0003
18	>=60 days since last violence	1.0054	0.9786	1.0330	0.6948
18	<60 days since last violence	1.0315	1.0137	1.0497	0.0005
19	>=60 days since last violence	1.0075	0.9811	1.0347	0.5809
19	<60 days since last violence	1.0308	1.0132	1.0487	0.0006
20	>=60 days since last violence	1.0066	0.9800	1.0340	0.6294
20	<60 days since last violence	1.0278	1.0097	1.0463	0.0025
21	>=60 days since last violence	1.0046	0.9778	1.0322	0.7387
21	<60 days since last violence	1.0275	1.0095	1.0458	0.0026
22	>=60 days since last violence	1.0050	0.9784	1.0324	0.7147
22	<60 days since last violence	1.0267	1.0086	1.0451	0.0037
23	>=60 days since last violence	1.0045	0.9771	1.0327	0.7491
23	<60 days since last violence	1.0266	1.0092	1.0442	0.0026
24	>=60 days since last violence	1.0044	0.9768	1.0328	0.7554
24	<60 days since last violence	1.0237	1.0068	1.0408	0.0057
25	>=60 days since last violence	1.0095	0.9823	1.0375	0.4984
25	<60 days since last violence	1.0216	1.0049	1.0386	0.0111
26	>=60 days since last violence	1.0071	0.9793	1.0357	0.6207
26	<60 days since last violence	1.0203	1.0030	1.0378	0.0210
27	>=60 days since last violence	1.0091	0.9821	1.0369	0.5112
27	<60 days since last violence	1.0196	1.0028	1.0366	0.0219
28	>=60 days since last violence	1.0058	0.9786	1.0338	0.6789
28	<60 days since last violence	1.0186	1.0023	1.0352	0.0253
29	>=60 days since last violence	1.0044	0.9764	1.0332	0.7593
29	<60 days since last violence	1.0204	1.0043	1.0367	0.0127
30	>=60 days since last violence	1.0059	0.9778	1.0348	0.6823
30	<60 days since last violence	1.0211	1.0048	1.0377	0.0111
31	>=60 days since last violence	1.0045	0.9752	1.0346	0.7682
31	<60 days since last violence	1.0227	1.0064	1.0393	0.0063
32	>=60 days since last violence	1.0048	0.9760	1.0343	0.7488

Continued on next page

Supplementary Table 6 – continued from previous page

Days after violence	Type of violence	Estimate	CI lower	CI upper	p-value
32	<60 days since last violence	1.0254	1.0091	1.0420	0.0021
33	>=60 days since last violence	1.0044	0.9752	1.0345	0.7692
33	<60 days since last violence	1.0271	1.0111	1.0434	0.0009
34	>=60 days since last violence	1.0066	0.9772	1.0370	0.6626
34	<60 days since last violence	1.0277	1.0119	1.0437	0.0005
35	>=60 days since last violence	1.0039	0.9746	1.0340	0.7976
35	<60 days since last violence	1.0292	1.0132	1.0454	0.0003
36	>=60 days since last violence	0.9984	0.9681	1.0296	0.9180
36	<60 days since last violence	1.0306	1.0154	1.0461	0.0001
37	>=60 days since last violence	0.9997	0.9707	1.0296	0.9824
37	<60 days since last violence	1.0305	1.0153	1.0459	0.0001
38	>=60 days since last violence	0.9995	0.9709	1.0288	0.9711
38	<60 days since last violence	1.0287	1.0134	1.0442	0.0002
39	>=60 days since last violence	0.9994	0.9707	1.0289	0.9658
39	<60 days since last violence	1.0283	1.0131	1.0437	0.0002
40	>=60 days since last violence	0.9971	0.9689	1.0261	0.8422
40	<60 days since last violence	1.0286	1.0138	1.0436	0.0001
41	>=60 days since last violence	0.9962	0.9679	1.0253	0.7954
41	<60 days since last violence	1.0293	1.0138	1.0450	0.0002
42	>=60 days since last violence	0.9954	0.9671	1.0245	0.7519
42	<60 days since last violence	1.0278	1.0123	1.0436	0.0004
43	>=60 days since last violence	0.9956	0.9674	1.0247	0.7662
43	<60 days since last violence	1.0267	1.0115	1.0422	0.0005
44	>=60 days since last violence	0.9932	0.9650	1.0222	0.6413
44	<60 days since last violence	1.0258	1.0105	1.0414	0.0009
45	>=60 days since last violence	0.9920	0.9639	1.0209	0.5851
45	<60 days since last violence	1.0257	1.0102	1.0414	0.0011
46	>=60 days since last violence	0.9924	0.9643	1.0213	0.6020
46	<60 days since last violence	1.0243	1.0089	1.0399	0.0019
47	>=60 days since last violence	0.9902	0.9616	1.0196	0.5093
47	<60 days since last violence	1.0249	1.0094	1.0407	0.0016
48	>=60 days since last violence	0.9887	0.9588	1.0195	0.4673

Continued on next page

Supplementary Table 6 – continued from previous page

Days after violence	Type of violence	Estimate	CI lower	CI upper	p-value
48	<60 days since last violence	1.0252	1.0097	1.0409	0.0014
49	>=60 days since last violence	0.9875	0.9575	1.0185	0.4247
49	<60 days since last violence	1.0226	1.0076	1.0378	0.0030
50	>=60 days since last violence	0.9882	0.9588	1.0185	0.4416
50	<60 days since last violence	1.0228	1.0078	1.0382	0.0029
51	>=60 days since last violence	0.9875	0.9580	1.0180	0.4180
51	<60 days since last violence	1.0234	1.0079	1.0391	0.0029
52	>=60 days since last violence	0.9891	0.9594	1.0198	0.4816
52	<60 days since last violence	1.0222	1.0068	1.0379	0.0046
53	>=60 days since last violence	0.9903	0.9614	1.0200	0.5172
53	<60 days since last violence	1.0228	1.0072	1.0386	0.0039
54	>=60 days since last violence	0.9895	0.9612	1.0187	0.4782
54	<60 days since last violence	1.0218	1.0062	1.0377	0.0061
55	>=60 days since last violence	0.9895	0.9605	1.0193	0.4849
55	<60 days since last violence	1.0207	1.0048	1.0369	0.0105
56	>=60 days since last violence	0.9864	0.9572	1.0164	0.3701
56	<60 days since last violence	1.0212	1.0049	1.0377	0.0106
57	>=60 days since last violence	0.9853	0.9559	1.0156	0.3369
57	<60 days since last violence	1.0218	1.0056	1.0383	0.0080
58	>=60 days since last violence	0.9857	0.9564	1.0160	0.3513
58	<60 days since last violence	1.0217	1.0059	1.0378	0.0070
59	>=60 days since last violence	0.9848	0.9556	1.0148	0.3169
59	<60 days since last violence	1.0217	1.0056	1.0381	0.0082
60	>=60 days since last violence	0.9851	0.9560	1.0152	0.3281
60	<60 days since last violence	1.0198	1.0038	1.0361	0.0153
61	>=60 days since last violence	0.9854	0.9557	1.0161	0.3483
61	<60 days since last violence	1.0196	1.0034	1.0361	0.0174
62	>=60 days since last violence	0.9858	0.9560	1.0166	0.3631
62	<60 days since last violence	1.0190	1.0035	1.0347	0.0159
63	>=60 days since last violence	0.9854	0.9545	1.0173	0.3653
63	<60 days since last violence	1.0202	1.0054	1.0352	0.0073
64	>=60 days since last violence	0.9872	0.9559	1.0196	0.4345

Continued on next page

Supplementary Table 6 – continued from previous page

Days after violence	Type of violence	Estimate	CI lower	CI upper	p-value
64	<60 days since last violence	1.0204	1.0059	1.0352	0.0057
65	>=60 days since last violence	0.9879	0.9561	1.0208	0.4660
65	<60 days since last violence	1.0209	1.0064	1.0356	0.0047
66	>=60 days since last violence	0.9905	0.9593	1.0227	0.5584
66	<60 days since last violence	1.0205	1.0062	1.0349	0.0047
67	>=60 days since last violence	0.9893	0.9580	1.0216	0.5110
67	<60 days since last violence	1.0210	1.0066	1.0356	0.0042
68	>=60 days since last violence	0.9894	0.9581	1.0217	0.5155
68	<60 days since last violence	1.0189	1.0040	1.0340	0.0129
69	>=60 days since last violence	0.9900	0.9587	1.0223	0.5403
69	<60 days since last violence	1.0178	1.0026	1.0333	0.0217
70	>=60 days since last violence	0.9874	0.9562	1.0197	0.4397
70	<60 days since last violence	1.0155	1.0000	1.0312	0.0504
71	>=60 days since last violence	0.9874	0.9561	1.0197	0.4405
71	<60 days since last violence	1.0160	1.0009	1.0313	0.0377
72	>=60 days since last violence	0.9881	0.9569	1.0204	0.4660
72	<60 days since last violence	1.0152	1.0006	1.0300	0.0411
73	>=60 days since last violence	0.9883	0.9571	1.0205	0.4716
73	<60 days since last violence	1.0149	1.0005	1.0296	0.0430
74	>=60 days since last violence	0.9870	0.9555	1.0195	0.4278
74	<60 days since last violence	1.0154	1.0014	1.0297	0.0310
75	>=60 days since last violence	0.9863	0.9546	1.0191	0.4099
75	<60 days since last violence	1.0171	1.0031	1.0313	0.0165
76	>=60 days since last violence	0.9863	0.9544	1.0191	0.4082
76	<60 days since last violence	1.0166	1.0030	1.0305	0.0168
77	>=60 days since last violence	0.9868	0.9548	1.0200	0.4320
77	<60 days since last violence	1.0164	1.0029	1.0301	0.0174
78	>=60 days since last violence	0.9873	0.9551	1.0205	0.4487
78	<60 days since last violence	1.0177	1.0038	1.0318	0.0124
79	>=60 days since last violence	0.9883	0.9559	1.0218	0.4888
79	<60 days since last violence	1.0180	1.0043	1.0319	0.0097
80	>=60 days since last violence	0.9897	0.9575	1.0229	0.5369

Continued on next page

Supplementary Table 6 – continued from previous page

Days after violence	Type of violence	Estimate	CI lower	CI upper	p-value
80	<60 days since last violence	1.0200	1.0068	1.0333	0.0028
81	>=60 days since last violence	0.9898	0.9574	1.0232	0.5432
81	<60 days since last violence	1.0200	1.0069	1.0332	0.0027
82	>=60 days since last violence	0.9917	0.9594	1.0250	0.6201
82	<60 days since last violence	1.0210	1.0078	1.0342	0.0017
83	>=60 days since last violence	0.9913	0.9594	1.0242	0.5980
83	<60 days since last violence	1.0219	1.0084	1.0356	0.0014
84	>=60 days since last violence	0.9926	0.9607	1.0256	0.6560
84	<60 days since last violence	1.0218	1.0082	1.0357	0.0017
85	>=60 days since last violence	0.9936	0.9618	1.0264	0.6966
85	<60 days since last violence	1.0236	1.0091	1.0383	0.0013
86	>=60 days since last violence	0.9932	0.9613	1.0262	0.6843
86	<60 days since last violence	1.0234	1.0090	1.0381	0.0014
87	>=60 days since last violence	0.9915	0.9594	1.0246	0.6091
87	<60 days since last violence	1.0233	1.0093	1.0376	0.0011
88	>=60 days since last violence	0.9925	0.9601	1.0259	0.6556
88	<60 days since last violence	1.0241	1.0103	1.0381	0.0006
89	>=60 days since last violence	0.9915	0.9591	1.0250	0.6147
89	<60 days since last violence	1.0238	1.0100	1.0378	0.0007
90	>=60 days since last violence	0.9919	0.9598	1.0252	0.6306
90	<60 days since last violence	1.0236	1.0103	1.0371	0.0005
91	>=60 days since last violence	0.9930	0.9607	1.0264	0.6786
91	<60 days since last violence	1.0216	1.0085	1.0349	0.0012
92	>=60 days since last violence	0.9929	0.9602	1.0268	0.6779
92	<60 days since last violence	1.0218	1.0088	1.0351	0.0010
93	>=60 days since last violence	0.9928	0.9598	1.0270	0.6770
93	<60 days since last violence	1.0204	1.0076	1.0334	0.0017
94	>=60 days since last violence	0.9942	0.9610	1.0285	0.7356
94	<60 days since last violence	1.0212	1.0081	1.0345	0.0014
95	>=60 days since last violence	0.9924	0.9591	1.0270	0.6638
95	<60 days since last violence	1.0210	1.0079	1.0342	0.0016
96	>=60 days since last violence	0.9923	0.9585	1.0273	0.6621

Continued on next page

Supplementary Table 6 – continued from previous page

Days after violence	Type of violence	Estimate	CI lower	CI upper	p-value
96	<60 days since last violence	1.0212	1.0081	1.0344	0.0014
97	>=60 days since last violence	0.9934	0.9592	1.0289	0.7120
97	<60 days since last violence	1.0225	1.0093	1.0360	0.0008
98	>=60 days since last violence	0.9906	0.9556	1.0269	0.6078
98	<60 days since last violence	1.0220	1.0089	1.0354	0.0010
99	>=60 days since last violence	0.9901	0.9547	1.0269	0.5938
99	<60 days since last violence	1.0223	1.0092	1.0356	0.0008
100	>=60 days since last violence	0.9886	0.9531	1.0255	0.5395
100	<60 days since last violence	1.0241	1.0107	1.0377	0.0004
101	>=60 days since last violence	0.9896	0.9539	1.0268	0.5796
101	<60 days since last violence	1.0243	1.0109	1.0379	0.0004
102	>=60 days since last violence	0.9886	0.9525	1.0261	0.5462
102	<60 days since last violence	1.0235	1.0101	1.0372	0.0006
103	>=60 days since last violence	0.9875	0.9507	1.0258	0.5186
103	<60 days since last violence	1.0239	1.0105	1.0374	0.0004
104	>=60 days since last violence	0.9880	0.9513	1.0260	0.5305
104	<60 days since last violence	1.0235	1.0100	1.0371	0.0006
105	>=60 days since last violence	0.9887	0.9520	1.0269	0.5577
105	<60 days since last violence	1.0238	1.0104	1.0373	0.0004
106	>=60 days since last violence	0.9892	0.9524	1.0273	0.5731
106	<60 days since last violence	1.0237	1.0100	1.0376	0.0007
107	>=60 days since last violence	0.9887	0.9520	1.0269	0.5581
107	<60 days since last violence	1.0237	1.0099	1.0376	0.0007
108	>=60 days since last violence	0.9887	0.9521	1.0268	0.5566
108	<60 days since last violence	1.0227	1.0092	1.0364	0.0010
109	>=60 days since last violence	0.9898	0.9532	1.0277	0.5921
109	<60 days since last violence	1.0225	1.0088	1.0364	0.0012
110	>=60 days since last violence	0.9921	0.9564	1.0292	0.6718
110	<60 days since last violence	1.0245	1.0101	1.0390	0.0008
111	>=60 days since last violence	0.9932	0.9577	1.0301	0.7140
111	<60 days since last violence	1.0254	1.0110	1.0400	0.0005
112	>=60 days since last violence	0.9933	0.9574	1.0305	0.7193

Continued on next page

Supplementary Table 6 – continued from previous page

Days after violence	Type of violence	Estimate	CI lower	CI upper	p-value
112	<60 days since last violence	1.0226	1.0084	1.0371	0.0018
113	>=60 days since last violence	0.9928	0.9569	1.0300	0.6995
113	<60 days since last violence	1.0220	1.0077	1.0365	0.0025
114	>=60 days since last violence	0.9905	0.9539	1.0285	0.6192
114	<60 days since last violence	1.0223	1.0079	1.0370	0.0024
115	>=60 days since last violence	0.9901	0.9535	1.0282	0.6069
115	<60 days since last violence	1.0222	1.0076	1.0371	0.0028
116	>=60 days since last violence	0.9905	0.9539	1.0285	0.6204
116	<60 days since last violence	1.0226	1.0079	1.0376	0.0026
117	>=60 days since last violence	0.9905	0.9539	1.0285	0.6195
117	<60 days since last violence	1.0215	1.0075	1.0356	0.0024
118	>=60 days since last violence	0.9892	0.9525	1.0273	0.5726
118	<60 days since last violence	1.0208	1.0068	1.0350	0.0035
119	>=60 days since last violence	0.9888	0.9524	1.0267	0.5575
119	<60 days since last violence	1.0198	1.0058	1.0340	0.0055
120	>=60 days since last violence	0.9919	0.9561	1.0290	0.6632
120	<60 days since last violence	1.0203	1.0061	1.0348	0.0050

Supplementary Table 7: Full model results for Figure 3d.

Days after violence	Type of violence	Estimate	CI lower	CI upper	p-value
1	Provincial capital	1.0198	0.9880	1.0527	0.2249
1	Non-capital	1.0250	0.9969	1.0539	0.0812
2	Provincial capital	1.0103	0.9808	1.0407	0.4980
2	Non-capital	1.0208	0.9999	1.0421	0.0512
3	Provincial capital	1.0135	0.9880	1.0396	0.3030
3	Non-capital	1.0346	1.0157	1.0538	0.0003
4	Provincial capital	1.0229	1.0012	1.0451	0.0388
4	Non-capital	1.0385	1.0215	1.0558	0.0000
5	Provincial capital	1.0126	0.9901	1.0355	0.2744
5	Non-capital	1.0372	1.0197	1.0551	0.0000
6	Provincial capital	1.0126	0.9910	1.0346	0.2563
6	Non-capital	1.0413	1.0229	1.0600	0.0000
7	Provincial capital	1.0098	0.9903	1.0296	0.3290
7	Non-capital	1.0443	1.0263	1.0627	0.0000
8	Provincial capital	1.0063	0.9875	1.0255	0.5137
8	Non-capital	1.0477	1.0302	1.0655	0.0000
9	Provincial capital	1.0099	0.9933	1.0268	0.2455
9	Non-capital	1.0481	1.0298	1.0666	0.0000
10	Provincial capital	1.0096	0.9939	1.0255	0.2336
10	Non-capital	1.0489	1.0312	1.0669	0.0000
11	Provincial capital	1.0081	0.9939	1.0225	0.2638
11	Non-capital	1.0485	1.0302	1.0672	0.0000
12	Provincial capital	1.0065	0.9925	1.0206	0.3677
12	Non-capital	1.0463	1.0277	1.0652	0.0000
13	Provincial capital	1.0058	0.9919	1.0198	0.4176
13	Non-capital	1.0434	1.0258	1.0613	0.0000
14	Provincial capital	1.0035	0.9902	1.0170	0.6048
14	Non-capital	1.0445	1.0269	1.0624	0.0000
15	Provincial capital	1.0022	0.9888	1.0157	0.7506
15	Non-capital	1.0433	1.0260	1.0610	0.0000
16	Provincial capital	1.0018	0.9888	1.0150	0.7827

Continued on next page

Supplementary Table 7 – continued from previous page

Days after violence	Type of violence	Estimate	CI lower	CI upper	p-value
16	Non-capital	1.0414	1.0236	1.0595	0.0000
17	Provincial capital	1.0004	0.9878	1.0131	0.9552
17	Non-capital	1.0394	1.0208	1.0584	0.0000
18	Provincial capital	1.0000	0.9880	1.0122	0.9956
18	Non-capital	1.0395	1.0206	1.0587	0.0000
19	Provincial capital	0.9995	0.9871	1.0121	0.9395
19	Non-capital	1.0404	1.0219	1.0592	0.0000
20	Provincial capital	0.9998	0.9871	1.0128	0.9805
20	Non-capital	1.0369	1.0182	1.0560	0.0001
21	Provincial capital	0.9995	0.9863	1.0129	0.9446
21	Non-capital	1.0359	1.0173	1.0548	0.0001
22	Provincial capital	0.9956	0.9828	1.0086	0.5039
22	Non-capital	1.0363	1.0178	1.0552	0.0001
23	Provincial capital	0.9955	0.9828	1.0084	0.4950
23	Non-capital	1.0355	1.0173	1.0540	0.0001
24	Provincial capital	0.9949	0.9826	1.0073	0.4199
24	Non-capital	1.0329	1.0147	1.0514	0.0004
25	Provincial capital	0.9948	0.9829	1.0068	0.3929
25	Non-capital	1.0335	1.0149	1.0523	0.0004
26	Provincial capital	0.9940	0.9816	1.0065	0.3435
26	Non-capital	1.0314	1.0124	1.0508	0.0011
27	Provincial capital	0.9950	0.9830	1.0072	0.4220
27	Non-capital	1.0314	1.0129	1.0504	0.0008
28	Provincial capital	0.9956	0.9831	1.0082	0.4898
28	Non-capital	1.0288	1.0108	1.0472	0.0017
29	Provincial capital	0.9963	0.9841	1.0087	0.5586
29	Non-capital	1.0293	1.0116	1.0473	0.0011
30	Provincial capital	0.9974	0.9853	1.0097	0.6809
30	Non-capital	1.0300	1.0123	1.0480	0.0008
31	Provincial capital	0.9982	0.9855	1.0112	0.7891
31	Non-capital	1.0304	1.0124	1.0488	0.0009
32	Provincial capital	0.9977	0.9842	1.0113	0.7370

Continued on next page

Supplementary Table 7 – continued from previous page

Days after violence	Type of violence	Estimate	CI lower	CI upper	p-value
32	Non-capital	1.0331	1.0152	1.0512	0.0002
33	Provincial capital	0.9979	0.9839	1.0121	0.7692
33	Non-capital	1.0343	1.0167	1.0521	0.0001
34	Provincial capital	0.9989	0.9847	1.0133	0.8817
34	Non-capital	1.0354	1.0183	1.0529	0.0000
35	Provincial capital	0.9986	0.9841	1.0134	0.8560
35	Non-capital	1.0355	1.0183	1.0531	0.0000
36	Provincial capital	0.9978	0.9834	1.0125	0.7679
36	Non-capital	1.0343	1.0172	1.0517	0.0001
37	Provincial capital	0.9974	0.9829	1.0122	0.7324
37	Non-capital	1.0349	1.0182	1.0518	0.0000
38	Provincial capital	0.9970	0.9827	1.0115	0.6824
38	Non-capital	1.0331	1.0167	1.0497	0.0001
39	Provincial capital	0.9964	0.9829	1.0100	0.5991
39	Non-capital	1.0329	1.0167	1.0493	0.0001
40	Provincial capital	0.9959	0.9829	1.0091	0.5433
40	Non-capital	1.0324	1.0168	1.0482	0.0000
41	Provincial capital	0.9956	0.9827	1.0086	0.5063
41	Non-capital	1.0327	1.0169	1.0488	0.0000
42	Provincial capital	0.9952	0.9825	1.0082	0.4700
42	Non-capital	1.0311	1.0151	1.0474	0.0001
43	Provincial capital	0.9950	0.9823	1.0079	0.4447
43	Non-capital	1.0300	1.0141	1.0461	0.0002
44	Provincial capital	0.9938	0.9820	1.0058	0.3119
44	Non-capital	1.0284	1.0126	1.0445	0.0004
45	Provincial capital	0.9928	0.9818	1.0039	0.2055
45	Non-capital	1.0280	1.0120	1.0442	0.0006
46	Provincial capital	0.9917	0.9812	1.0024	0.1269
46	Non-capital	1.0272	1.0112	1.0434	0.0008
47	Provincial capital	0.9927	0.9826	1.0029	0.1598
47	Non-capital	1.0264	1.0109	1.0421	0.0008
48	Provincial capital	0.9940	0.9842	1.0038	0.2269

Continued on next page

Supplementary Table 7 – continued from previous page

Days after violence	Type of violence	Estimate	CI lower	CI upper	p-value
48	Non-capital	1.0258	1.0103	1.0416	0.0010
49	Provincial capital	0.9946	0.9853	1.0040	0.2555
49	Non-capital	1.0232	1.0082	1.0385	0.0024
50	Provincial capital	0.9963	0.9871	1.0055	0.4268
50	Non-capital	1.0231	1.0082	1.0382	0.0022
51	Provincial capital	0.9961	0.9866	1.0056	0.4163
51	Non-capital	1.0234	1.0083	1.0386	0.0022
52	Provincial capital	0.9952	0.9853	1.0052	0.3469
52	Non-capital	1.0234	1.0084	1.0387	0.0022
53	Provincial capital	0.9958	0.9862	1.0055	0.3939
53	Non-capital	1.0243	1.0095	1.0393	0.0012
54	Provincial capital	0.9962	0.9862	1.0063	0.4614
54	Non-capital	1.0229	1.0081	1.0380	0.0023
55	Provincial capital	0.9962	0.9859	1.0066	0.4724
55	Non-capital	1.0219	1.0069	1.0371	0.0040
56	Provincial capital	0.9952	0.9845	1.0059	0.3767
56	Non-capital	1.0212	1.0061	1.0367	0.0060
57	Provincial capital	0.9951	0.9840	1.0063	0.3924
57	Non-capital	1.0214	1.0063	1.0368	0.0053
58	Provincial capital	0.9947	0.9832	1.0063	0.3688
58	Non-capital	1.0217	1.0069	1.0367	0.0040
59	Provincial capital	0.9950	0.9831	1.0070	0.4125
59	Non-capital	1.0210	1.0060	1.0362	0.0060
60	Provincial capital	0.9948	0.9826	1.0073	0.4144
60	Non-capital	1.0196	1.0045	1.0349	0.0107
61	Provincial capital	0.9951	0.9828	1.0076	0.4436
61	Non-capital	1.0196	1.0044	1.0351	0.0114
62	Provincial capital	0.9955	0.9825	1.0087	0.5020
62	Non-capital	1.0194	1.0048	1.0342	0.0091
63	Provincial capital	0.9968	0.9836	1.0102	0.6397
63	Non-capital	1.0200	1.0057	1.0344	0.0060
64	Provincial capital	0.9976	0.9845	1.0108	0.7197

Continued on next page

Supplementary Table 7 – continued from previous page

Days after violence	Type of violence	Estimate	CI lower	CI upper	p-value
64	Non-capital	1.0208	1.0067	1.0351	0.0037
65	Provincial capital	0.9978	0.9843	1.0115	0.7553
65	Non-capital	1.0215	1.0075	1.0356	0.0025
66	Provincial capital	0.9986	0.9848	1.0126	0.8422
66	Non-capital	1.0219	1.0080	1.0361	0.0020
67	Provincial capital	0.9969	0.9827	1.0112	0.6666
67	Non-capital	1.0223	1.0083	1.0364	0.0017
68	Provincial capital	0.9961	0.9815	1.0109	0.6056
68	Non-capital	1.0211	1.0068	1.0355	0.0036
69	Provincial capital	0.9965	0.9817	1.0114	0.6415
69	Non-capital	1.0204	1.0062	1.0348	0.0047
70	Provincial capital	0.9972	0.9828	1.0119	0.7102
70	Non-capital	1.0167	1.0018	1.0318	0.0279
71	Provincial capital	0.9975	0.9838	1.0114	0.7232
71	Non-capital	1.0168	1.0020	1.0318	0.0255
72	Provincial capital	0.9975	0.9840	1.0111	0.7168
72	Non-capital	1.0163	1.0020	1.0308	0.0255
73	Provincial capital	0.9975	0.9843	1.0108	0.7094
73	Non-capital	1.0160	1.0019	1.0302	0.0262
74	Provincial capital	0.9987	0.9857	1.0120	0.8517
74	Non-capital	1.0154	1.0015	1.0295	0.0294
75	Provincial capital	0.9984	0.9850	1.0120	0.8190
75	Non-capital	1.0167	1.0031	1.0305	0.0161
76	Provincial capital	0.9986	0.9849	1.0124	0.8377
76	Non-capital	1.0163	1.0029	1.0299	0.0173
77	Provincial capital	0.9996	0.9855	1.0138	0.9510
77	Non-capital	1.0161	1.0029	1.0294	0.0168
78	Provincial capital	0.9997	0.9853	1.0144	0.9707
78	Non-capital	1.0172	1.0037	1.0308	0.0124
79	Provincial capital	1.0003	0.9858	1.0150	0.9663
79	Non-capital	1.0178	1.0045	1.0312	0.0085
80	Provincial capital	1.0014	0.9866	1.0163	0.8563

Continued on next page

Supplementary Table 7 – continued from previous page

Days after violence	Type of violence	Estimate	CI lower	CI upper	p-value
80	Non-capital	1.0200	1.0072	1.0329	0.0021
81	Provincial capital	1.0003	0.9855	1.0153	0.9659
81	Non-capital	1.0206	1.0077	1.0337	0.0017
82	Provincial capital	1.0001	0.9849	1.0156	0.9867
82	Non-capital	1.0227	1.0099	1.0357	0.0005
83	Provincial capital	0.9995	0.9842	1.0150	0.9469
83	Non-capital	1.0235	1.0103	1.0368	0.0005
84	Provincial capital	0.9990	0.9843	1.0138	0.8892
84	Non-capital	1.0243	1.0112	1.0376	0.0003
85	Provincial capital	0.9994	0.9850	1.0139	0.9321
85	Non-capital	1.0262	1.0127	1.0399	0.0001
86	Provincial capital	0.9992	0.9853	1.0132	0.9068
86	Non-capital	1.0260	1.0125	1.0397	0.0002
87	Provincial capital	0.9991	0.9857	1.0126	0.8942
87	Non-capital	1.0247	1.0114	1.0382	0.0002
88	Provincial capital	0.9991	0.9860	1.0123	0.8910
88	Non-capital	1.0260	1.0131	1.0389	0.0001
89	Provincial capital	0.9988	0.9859	1.0119	0.8615
89	Non-capital	1.0254	1.0124	1.0385	0.0001
90	Provincial capital	0.9997	0.9875	1.0120	0.9570
90	Non-capital	1.0251	1.0122	1.0382	0.0001
91	Provincial capital	0.9999	0.9877	1.0122	0.9817
91	Non-capital	1.0238	1.0106	1.0372	0.0004
92	Provincial capital	1.0003	0.9886	1.0122	0.9550
92	Non-capital	1.0239	1.0108	1.0372	0.0003
93	Provincial capital	1.0003	0.9886	1.0120	0.9647
93	Non-capital	1.0228	1.0097	1.0361	0.0006
94	Provincial capital	1.0007	0.9891	1.0124	0.9092
94	Non-capital	1.0240	1.0107	1.0374	0.0004
95	Provincial capital	1.0010	0.9897	1.0125	0.8602
95	Non-capital	1.0229	1.0096	1.0365	0.0007
96	Provincial capital	1.0018	0.9902	1.0135	0.7665

Continued on next page

Supplementary Table 7 – continued from previous page

Days after violence	Type of violence	Estimate	CI lower	CI upper	p-value
96	Non-capital	1.0230	1.0095	1.0367	0.0008
97	Provincial capital	1.0023	0.9913	1.0135	0.6802
97	Non-capital	1.0247	1.0110	1.0386	0.0004
98	Provincial capital	1.0025	0.9921	1.0130	0.6382
98	Non-capital	1.0229	1.0087	1.0374	0.0015
99	Provincial capital	1.0024	0.9923	1.0126	0.6453
99	Non-capital	1.0230	1.0087	1.0375	0.0016
100	Provincial capital	1.0034	0.9939	1.0130	0.4872
100	Non-capital	1.0234	1.0088	1.0381	0.0016
101	Provincial capital	1.0044	0.9954	1.0135	0.3412
101	Non-capital	1.0240	1.0093	1.0389	0.0013
102	Provincial capital	1.0043	0.9951	1.0136	0.3607
102	Non-capital	1.0228	1.0079	1.0378	0.0025
103	Provincial capital	1.0044	0.9952	1.0137	0.3512
103	Non-capital	1.0225	1.0075	1.0378	0.0032
104	Provincial capital	1.0047	0.9955	1.0140	0.3196
104	Non-capital	1.0221	1.0071	1.0373	0.0038
105	Provincial capital	1.0047	0.9952	1.0142	0.3320
105	Non-capital	1.0226	1.0075	1.0379	0.0032
106	Provincial capital	1.0057	0.9962	1.0153	0.2419
106	Non-capital	1.0223	1.0071	1.0379	0.0040
107	Provincial capital	1.0054	0.9961	1.0149	0.2551
107	Non-capital	1.0220	1.0067	1.0376	0.0047
108	Provincial capital	1.0053	0.9958	1.0149	0.2793
108	Non-capital	1.0210	1.0061	1.0362	0.0057
109	Provincial capital	1.0058	0.9957	1.0159	0.2592
109	Non-capital	1.0208	1.0059	1.0360	0.0062
110	Provincial capital	1.0061	0.9961	1.0162	0.2343
110	Non-capital	1.0232	1.0083	1.0383	0.0022
111	Provincial capital	1.0064	0.9964	1.0165	0.2129
111	Non-capital	1.0243	1.0093	1.0395	0.0014
112	Provincial capital	1.0069	0.9965	1.0174	0.1971

Continued on next page

Supplementary Table 7 – continued from previous page

Days after violence	Type of violence	Estimate	CI lower	CI upper	p-value
112	Non-capital	1.0215	1.0065	1.0368	0.0048
113	Provincial capital	1.0072	0.9962	1.0183	0.2023
113	Non-capital	1.0205	1.0055	1.0358	0.0072
114	Provincial capital	1.0077	0.9964	1.0192	0.1831
114	Non-capital	1.0196	1.0043	1.0352	0.0120
115	Provincial capital	1.0074	0.9959	1.0190	0.2064
115	Non-capital	1.0194	1.0036	1.0353	0.0156
116	Provincial capital	1.0071	0.9960	1.0184	0.2117
116	Non-capital	1.0196	1.0039	1.0356	0.0142
117	Provincial capital	1.0077	0.9963	1.0191	0.1859
117	Non-capital	1.0181	1.0030	1.0334	0.0187
118	Provincial capital	1.0079	0.9964	1.0196	0.1809
118	Non-capital	1.0167	1.0017	1.0319	0.0290
119	Provincial capital	1.0073	0.9957	1.0190	0.2205
119	Non-capital	1.0158	1.0009	1.0310	0.0378
120	Provincial capital	1.0078	0.9961	1.0196	0.1911
120	Non-capital	1.0173	1.0023	1.0325	0.0232

Supplementary Table 8: Full model results for Figure 4.

Variable	Days since violence	Estimate	CI lower	CI upper	p-value
IS	1-15	1.2730	1.0828	1.4965	0.0035
IS	16-30	1.2364	1.0765	1.4201	0.0027
IS	31-45	1.2820	1.1164	1.4721	0.0004
IS	46-60	1.3379	1.1248	1.5913	0.0010
IS	61-75	1.3194	1.1232	1.5498	0.0007
IS	76-90	1.2511	1.0615	1.4745	0.0075
High casualty	1-15	1.0242	0.9713	1.0799	0.3768
High casualty	16-30	1.0071	0.9670	1.0489	0.7329
High casualty	31-45	1.0041	0.9495	1.0618	0.8869
High casualty	46-60	1.0314	0.9747	1.0915	0.2837
High casualty	61-75	0.9909	0.9492	1.0345	0.6780
High casualty	76-90	0.9963	0.9236	1.0746	0.9227
Long peacetime	1-15	0.9537	0.9239	0.9845	0.0035
Long peacetime	16-30	0.9583	0.9291	0.9885	0.0071
Long peacetime	31-45	0.9489	0.9190	0.9797	0.0013
Long peacetime	46-60	0.9519	0.9234	0.9813	0.0015
Long peacetime	61-75	0.9741	0.9456	1.0034	0.0827
Long peacetime	76-90	0.9856	0.9572	1.0148	0.3306
Provincial capital	1-15	0.9924	0.9616	1.0242	0.6369
Provincial capital	16-30	0.9788	0.9469	1.0119	0.2063
Provincial capital	31-45	0.9863	0.9500	1.0240	0.4716
Provincial capital	46-60	0.9837	0.9403	1.0293	0.4775
Provincial capital	61-75	0.9940	0.9604	1.0288	0.7334
Provincial capital	76-90	0.9969	0.9636	1.0314	0.8585
Population (log)	1-15	1.0053	0.9865	1.0245	0.5832
Population (log)	16-30	0.9969	0.9804	1.0137	0.7157
Population (log)	31-45	0.9879	0.9690	1.0073	0.2203
Population (log)	46-60	0.9951	0.9752	1.0155	0.6368
Population (log)	61-75	1.0042	0.9857	1.0230	0.6570
Population (log)	76-90	1.0073	0.9901	1.0248	0.4070

Supplementary Table 9: Full model results for Figure 6.

Origin district	Destination district	Days after violence	Estimate	CI lower	CI upper	p-value
Non-capitals	Same province: Non-capital	7	1.0112	0.9808	1.0426	0.4732
Non-capitals	Same province: Non-capital	30	1.0078	0.9847	1.0314	0.5128
Non-capitals	Same province: Non-capital	90	1.0030	0.9842	1.0223	0.7539
Non-capitals	Same province: Capital	7	1.0262	0.9979	1.0554	0.0703
Non-capitals	Same province: Capital	30	1.0324	1.0142	1.0510	0.0005
Non-capitals	Same province: Capital	90	1.0229	1.0050	1.0410	0.0118
Non-capitals	Different province: Top-5 cities	7	0.9610	0.9367	0.9858	0.0023
Non-capitals	Different province: Top-5 cities	30	0.9730	0.9554	0.9909	0.0033
Non-capitals	Different province: Top-5 cities	90	0.9718	0.9577	0.9860	0.0001
Non-capitals	Different province: Other capitals	7	0.9848	0.9596	1.0106	0.2445
Non-capitals	Different province: Other capitals	30	0.9871	0.9677	1.0069	0.2002
Non-capitals	Different province: Other capitals	90	0.9969	0.9806	1.0135	0.7148
Non-capitals	Different province: Non-capital	7	0.9804	0.9530	1.0085	0.1697
Non-capitals	Different province: Non-capital	30	0.9855	0.9690	1.0022	0.0889
Non-capitals	Different province: Non-capital	90	0.9966	0.9827	1.0106	0.6276
Capitals	Same province: Non-capital	7	0.9756	0.9485	1.0034	0.0849
Capitals	Same province: Non-capital	30	0.9819	0.9635	1.0007	0.0590
Capitals	Same province: Non-capital	90	0.9967	0.9820	1.0115	0.6592
Capitals	Different province: Top-5 cities	7	1.0100	0.9943	1.0260	0.2129
Capitals	Different province: Top-5 cities	30	1.0063	0.9939	1.0190	0.3215
Capitals	Different province: Top-5 cities	90	1.0000	0.9847	1.0154	0.9980
Capitals	Different province: Other capitals	7	0.9945	0.9759	1.0135	0.5676
Capitals	Different province: Other capitals	30	1.0011	0.9878	1.0145	0.8763
Capitals	Different province: Other capitals	90	1.0051	0.9864	1.0242	0.5947
Capitals	Different province: Non-capital	7	1.0063	0.9837	1.0293	0.5877
Capitals	Different province: Non-capital	30	1.0094	0.9985	1.0205	0.0916
Capitals	Different province: Non-capital	90	1.0039	0.9886	1.0195	0.6159
