# Supplementary material for: Mobile phone data reveal the effects of violence on internal displacement in Afghanistan
Source: Nat Hum Behav. 2022 May 12;6(5):624–34. doi: 10.1038/s41562-022-01336-4 (PMC9130096; doi:10.1038/s41562-022-01336-4)
Supplement: Supplementary file 2 — Reporting Summary. [file 41562_2022_1336_MOESM2_ESM.pdf]

nature research

Corresponding author(s): Joshua Blumenstock

Last updated by author(s): Mar 3, 2022

Reporting Summary

Nature Research wishes to improve the reproducibility of the work that we publish. This form provides structure for consistency and transparency in reporting. For further information on Nature Research policies, see our Editorial Policies and the Editorial Policy Checklist.

Statistics

For all statistical analyses, confirm that the following items are present in the figure legend, table legend, main text, or Methods section.

n/a	Confirmed	
☐	☒	The exact sample size (*n*) for each experimental group/condition, given as a discrete number and unit of measurement
☐	☒	A statement on whether measurements were taken from distinct samples or whether the same sample was measured repeatedly
☐	☒	The statistical test(s) used AND whether they are one- or two-sided<br>*Only common tests should be described solely by name; describe more complex techniques in the Methods section.*
☐	☒	A description of all covariates tested
☐	☒	A description of any assumptions or corrections, such as tests of normality and adjustment for multiple comparisons
☐	☒	A full description of the statistical parameters including central tendency (e.g. means) or other basic estimates (e.g. regression coefficient) AND variation (e.g. standard deviation) or associated estimates of uncertainty (e.g. confidence intervals)
☐	☒	For null hypothesis testing, the test statistic (e.g. *F*, *t*, *r*) with confidence intervals, effect sizes, degrees of freedom and *P* value noted<br>*Give P values as exact values whenever suitable.*
☒	☐	For Bayesian analysis, information on the choice of priors and Markov chain Monte Carlo settings
☒	☐	For hierarchical and complex designs, identification of the appropriate level for tests and full reporting of outcomes
☐	☒	Estimates of effect sizes (e.g. Cohen's *d*, Pearson's *r*), indicating how they were calculated

Our web collection on statistics for biologists contains articles on many of the points above.

Software and code

Policy information about availability of computer code

Data collection	All code to reproduce the findings of this study is available at https://github.com/shikharmehra/afghanistan-internal-displacement.
Data analysis	All code to reproduce the findings of this study is available at https://github.com/shikharmehra/afghanistan-internal-displacement. We use Python 3.9.1, R 4.1.2, and custom Python package migration_detector v0.1.2 (https://github.com/g-chi/migration_detector). No other non-standard libraries were used.

For manuscripts utilizing custom algorithms or software that are central to the research but not yet described in published literature, software must be made available to editors and reviewers. We strongly encourage code deposition in a community repository (e.g. GitHub). See the Nature Research guidelines for submitting code & software for further information.

Data

Policy information about availability of data

All manuscripts must include a data availability statement. This statement should provide the following information, where applicable:

- Accession codes, unique identifiers, or web links for publicly available datasets
- A list of figures that have associated raw data
- A description of any restrictions on data availability

The mobile phone dataset contains detailed information on roughly 20 billion mobile phone transactions in Afghanistan. These data contain proprietary and confidential information belonging to a private telecommunications operator, and cannot be publicly released. Upon reasonable request, we can provide information to accredited academic researchers about how to request the proprietary data from the telecommunications operator. With the telecommunication operator's permission, we can also provide district-level aggregate measures of migration for replication purposes to accredited academic researchers. All other

data used in this paper are publicly available and sources have been listed in the text. They are: UCDP Georeferenced Event Dataset (GED) Global version 19.1 (https://ucdp.uu.se/downloads/), Afghanistan district level population data (https://data.humdata.org/dataset/estimated-population-of-afghanistan-2015-2016), displacement measures published by the IOM (DTM Afghanistan Districts Round 9 Baseline Assessment, available at https://data.humdata.org/dataset/afghanistan-displacement-data-baseline-assessment-iom-dtm). Shape files for Afghanistan are available at https://esoc.princeton.edu/data/administrative-boundaries-398-districts.

Field-specific reporting

Please select the one below that is the best fit for your research. If you are not sure, read the appropriate sections before making your selection.

☐ Life sciences ☒ Behavioural & social sciences ☐ Ecological, evolutionary & environmental sciences

For a reference copy of the document with all sections, see nature.com/documents/nr-reporting-summary-flat.pdf

Behavioural & social sciences study design

All studies must disclose on these points even when the disclosure is negative.

Study description	This is a quantitative study using mobile phone metadata.
Research sample	The dataset is collected from subscribers of Afghanistan's largest mobile phone operator, who made at least one call or recorded data use in the period April 2013 to March 2017. This contains roughly 20 billion mobile phone transactions and were collected by a private telecommunications operator. This dataset is not representative of the full Afghan population (but is representative of subscribers on this network).
Sampling strategy	All available data are used.
Data collection	Mobile phone data were collected by Afghanistan's largest mobile phone operator using their proprietary systems. Violent events data were collected from media reports and publicly released by the Uppsala Conflict Data Program.
Timing	Data are available from April 2013 to March 2017.
Data exclusions	No data were excluded from the analysis.
Non-participation	No participants were involved the study.
Randomization	This is a study using observational data, and no randomization was done.

Reporting for specific materials, systems and methods

We require information from authors about some types of materials, experimental systems and methods used in many studies. Here, indicate whether each material, system or method listed is relevant to your study. If you are not sure if a list item applies to your research, read the appropriate section before selecting a response.

Materials & experimental systems

n/a	Involved in the study	
☒	☐	Antibodies
☒	☐	Eukaryotic cell lines
☒	☐	Palaeontology and archaeology
☒	☐	Animals and other organisms
☒	☐	Human research participants
☒	☐	Clinical data
☒	☐	Dual use research of concern

Methods

n/a	Involved in the study	
☒	☐	ChIP-seq
☒	☐	Flow cytometry
☒	☐	MRI-based neuroimaging
